# A new family of CRISPR‐type V nucleases with C‐rich PAM recognition

**DOI:** 10.15252/embr.202255481

**Published:** 2022-10-21

**Authors:** Tomas Urbaitis, Giedrius Gasiunas, Joshua K Young, Zhenglin Hou, Sushmitha Paulraj, Egle Godliauskaite, Mantvyda M Juskeviciene, Migle Stitilyte, Monika Jasnauskaite, Megumu Mabuchi, G Brett Robb, Virginijus Siksnys

**Affiliations:** ^1^ CasZyme Vilnius Lithuania; ^2^ Institute of Biotechnology Vilnius University Vilnius Lithuania; ^3^ Genomics Technologies Corteva Agriscience™ Johnston IA USA; ^4^ Farming Solutions & Digital Corteva Agriscience™ Johnston IA USA; ^5^ New England Biolabs Ipswich MA USA; ^6^ Present address: LSC‐EMBL Partnership Institute for Genome Technologies Editing, Life Sciences Center Vilnius University Vilnius Lithuania

**Keywords:** CRISPR‐Cas, genome editing, nucleic acid detection, PAM, RNA‐guided nuclease, Chromatin, Transcription & Genomics, Methods & Resources

## Abstract

Most CRISPR‐type V nucleases are stimulated to cleave double‐stranded (ds) DNA targets by a T‐rich PAM, which restricts their targeting range. Here, we identify and characterize a new family of type V RNA‐guided nuclease, Cas12l, that exclusively recognizes a C‐rich (5'‐CCY‐3′) PAM. The organization of genes within its CRISPR locus is similar to type II‐B CRISPR‐Cas9 systems, but both sequence analysis and functional studies establish it as a new family of type V effector. Biochemical experiments show that Cas12l nucleases function optimally between 37 and 52°C, depending on the ortholog, and preferentially cut supercoiled DNA. Like other type V nucleases, it exhibits collateral nonspecific ssDNA and ssRNA cleavage activity that is triggered by ssDNA or dsDNA target recognition. Finally, we show that one family member, Asp2Cas12l, functions in a heterologous cellular environment, altogether, suggesting that this new group of CRISPR‐associated nucleases may be harnessed as genome editing reagents.

## Graphical abstract

Cas12l is a new family of type V CRISPR‐Cas nucleases that recognizes a C‐rich PAM and potentially expands our genome editing toolbox.

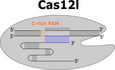

## Introduction

Archaea and bacteria use clustered regularly interspaced short palindromic repeat (CRISPR) systems coupled with CRISPR‐associated (Cas) proteins as an adaptive immune system against invading nucleic acids (Barrangou & Marraffini, 2014). The CRISPR array serves as a template to produce CRISPR RNAs (crRNAs) that harbor spacer sequences acquired from foreign genetic elements (Bolotin *et al*, 2005; Barrangou *et al*, 2007; Brouns *et al*, 2008; Hale *et al*, 2008). crRNAs then guide Cas nucleases to cleave DNA and RNAs with base‐pair complementarity to the spacer sequences (Hale *et al*, 2009; Garneau *et al*, 2010; Jore *et al*, 2011). Based on the number and composition of Cas proteins involved in nucleic acid interference, CRISPR‐Cas systems are categorized into 2 classes and 6 types (I‐VI; Makarova *et al*, 2020). Class 2 systems require only a single effector protein for nucleic acid cleavage and are further subdivided into 3 types (II, V, and VI). Type II (Cas9) and V (Cas12) effector nucleases use their guide RNA to recognize and cleave dsDNA targets close to a short sequence motif termed the protospacer adjacent motif (PAM) (Gasiunas *et al*, 2012; Jinek *et al*, 2012; Zetsche *et al*, 2015). In addition to a crRNA, some class 2 systems require an additional RNA molecule encoded in the CRISPR locus called a trans‐activating RNA (tracrRNA) to function (Deltcheva *et al*, 2011). In these systems, the crRNA and tracrRNA can be linked together into a single guide RNA (sgRNA; Jinek *et al*, 2012). Over the past years, Cas9 and Cas12a proteins have been harnessed as versatile gene editing tools and their biochemical attributes successfully adapted to detect nucleic acids (Cong *et al*, 2013; Mali *et al*, 2013; Wang *et al*, 2020).

While Cas9 orthologs form an exceptionally diverse family, protein architecture and length (~1,000–1,600 aa) remain similar (Fonfara *et al*, 2014; Gasiunas *et al*, 2020). By contrast, the size of type V effectors is more divergent (Shmakov *et al*, 2017). In this case, nucleases range between ~400 and 1,500 aa and have been shown to be structurally distinct (Liu *et al*, 2017, 2019; Takeda *et al*, 2021). Due to this rich variation, CRISPR‐Cas12 nucleases continue to be mined and developed for use as genome editing tools. This includes the discovery, characterization, and optimization of compact nucleases that offer to simplify delivery by easing viral genome size packaging constraints (Karvelis *et al*, 2020; Pausch *et al*, 2020; Bigelyte *et al*, 2021; Kim *et al*, 2021; Wu *et al*, 2021; Xu *et al*, 2021). Despite these efforts, most Cas12 proteins described to date recognize a T‐rich PAM, which restricts their targeting range (Shmakov *et al*, 2015; Zetsche *et al*, 2015; Burstein *et al*, 2017; Karvelis *et al*, 2020; Pausch *et al*, 2020). This becomes particularly apparent in situations that require genomes or regions with elevated GC content to be targeted. These limitations may be further compounded in genome editing applications where the outcome is dependent on the proximity of the desired edit to the cut site (e.g., template‐free editing and homology‐directed repair) or for approaches that impose additional sequence requirements on target selection (e.g., base editing; Anzalone *et al*, 2020).

In this study, we identify and characterize a new family of type V CRISPR nuclease from a relatively unexplored phylum of bacteria, *Armatimonadetes*. The locus gene architecture is reminiscent of type II‐B Cas9 systems and encodes a single effector nuclease followed by *cas1*, *cas2*, and *cas4* genes and the CRISPR array. The effector nuclease encoded in the locus is compact being ~860 aa. Through biochemical experimentation, we show that family members exclusively require a 5' C‐rich PAM to license dsDNA cleavage. *In vitro* experiments with purified components demonstrate that DNA substrate topology affects cleavage activity with supercoiled targets being preferred over relaxed ones. Target recognition is also shown to trigger nonspecific collateral ssDNA or ssRNA degradation at rates equivalent to Cas12a. Finally, we show that one member of the family, Asp2Cas12l, functions in *Escherichia coli* to protect against “invading” plasmid DNA, altogether, suggesting that this new family of effector nucleases may be harnessed for genome editing applications.

## Results

### Identification of CRISPR‐Cas12l systems

**Figure 1:**
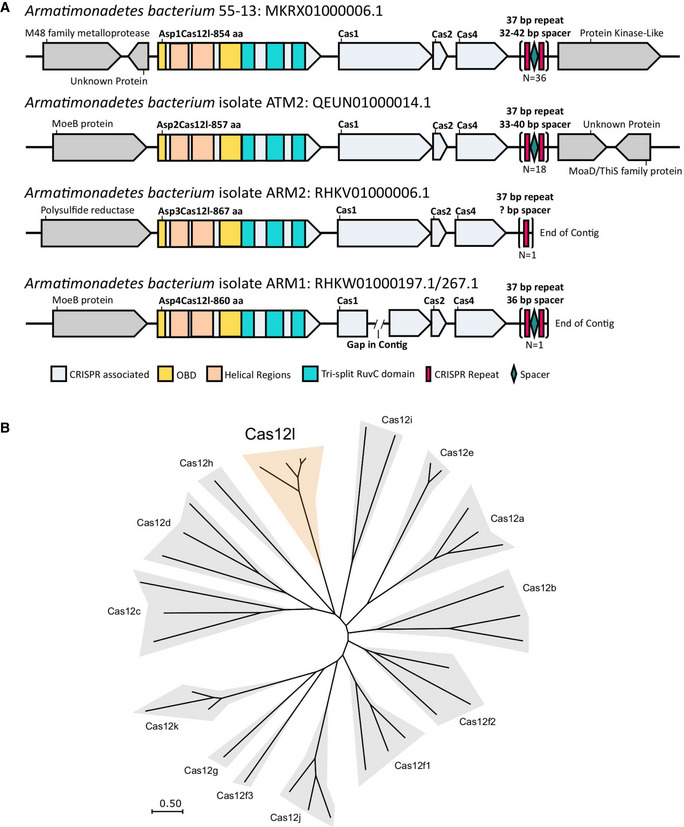
Cas12l represents a new family of type V CRISPR‐associated nuclease A schematic of Cas12l locus architecture. CRISPR‐associated genes are shown in light blue and each locus identified encodes one approximately 860 aa effector containing a single tri‐split RuvC nuclease domain and a helix‐turn‐helix motif (orange) followed by Cas1, Cas2, and Cas4.Maximum likelihood phylogenetic tree illustrating the sequence relationship between Cas12l and other Type V CRISPR‐Cas proteins.

New type V CRISPR‐Cas systems were identified by searching microbial sequence datasets for CRISPR‐associated nucleases that contain a single RuvC domain encoded in an operon‐like organization with *cas1* and *cas2* genes. CRISPR locus gene architecture, structural inspection of the putative effector nuclease, and phylogenetic analysis were then used to define new systems. Using this methodology, a new family of type V CRISPR‐associated nuclease was discovered in species of *Armatimonadetes* whose sequence was captured in metagenomic studies aimed at identifying microbial communities involved in wastewater treatment (Kantor *et al*, 2017; Zhao *et al*, 2018). Contigs for the first three CRISPR‐Cas systems were contiguous with the CRISPR array while two fragments were identified as belonging to the fourth (Fig 1A). In each locus were genes that together encoded proteins required for adaptation (Cas1, Cas2, and Cas4) and a compact (~860 aa) effector nuclease adjacent to a CRISPR array (Fig 1A). The positioning and orientation of the putative effector and adaptation genes within the CRISPR locus (*nuclease*, *cas1*, *cas2*, *cas4*, and CRISPR array) resembled that of type II‐B CRISPR‐Cas9 systems (Koonin & Makarova, 2019; Fig 1A). Sequence examination of the putative nuclease confirmed the presence of a single tri‐split RuvC‐like domain located in the C‐terminal half of the protein similar to other Cas12 proteins (Zetsche *et al*, 2015; Shmakov *et al*, 2017) (Fig 1A). By contrast, the sequence of the N‐terminal half was highly divergent from other Cas12 nucleases. Despite this, it could be predicted to form an oligo binding domain (OBD) split by two helical regions containing a bridge‐helix (BH)‐like motif and a helix‐turn‐helix (HTH) DNA binding domain (Fig 1A). Phylogenetic analysis of the nuclease family confirmed their classification as a type V effector and showed that they form a new subgroup distinct from previously described Cas12 proteins (Fig 1B). To simplify nomenclature and align with CRISPR nuclease naming conventions, we propose they be classified as Cas12l.

### CRISPR‐Cas12l utilizes a single effector nuclease to cleave dsDNA

**Figure 2:**
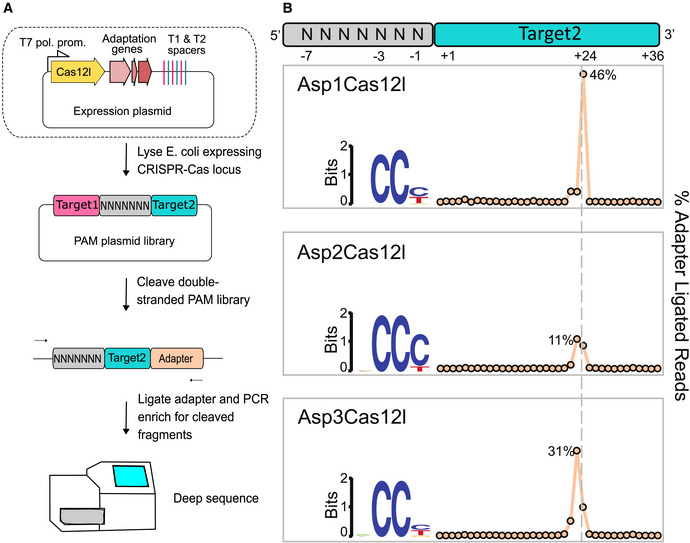
Cas12l nucleases cleave double‐stranded (ds) DNA in the presence of a 5' C‐rich PAM Workflow used to detect dsDNA cleavage and associated PAM recognition of Cas12l CRISPR systems. *E. coli* was transformed with plasmids encoding an intact CRISPR locus encoding the Cas12l effector nuclease and adaptation proteins, Cas1, Cas2, and Cas4, engineered to target a randomized PAM plasmid library (T1 and T2 spacers correspond to Target1 and Target2). After expression, cells were disrupted and the resulting lysate used in subsequent steps. Cleavage products were captured by adapter ligation, enriched for using PCR, and subjected to Illumina deep sequencing.dsDNA cleavage was detected by examining the frequency of adapter ligation at each position of the protospacer target relative to control reactions assembled with an empty vector. Once identified, reads associated with the highest frequency of adapter ligation were used to assess PAM recognition. Weblogos of the PAM sequences that supported target recognition and cleavage are shown.

To determine whether Cas12l systems were capable of dsDNA hydrolysis, lysate from *E. coli* expressing Cas12l proteins and guide RNAs were used to interrogate a 7N randomized PAM library similar to that described earlier (Karvelis *et al*, 2020; Fig 2A). This was accomplished by first modifying Cas12l CRISPR arrays to encode spacer sequences capable of targeting either side of the PAM library (Fig 2A). This design allowed for the capture of both possible PAM orientations, 5′ and 3′, when characterizing new CRISPR‐Cas systems (Fig 2A). Spacer altered Cas12l CRISPR systems were then synthesized and cloned into IPTG inducible expression plasmids and transformed into *E. coli*. After inducing expression, cells were disrupted, and the clarified lysate was combined with the PAM library. Cleavage products were then captured by dsDNA adapter ligation, enriched for by PCR, and subjected to Illumina deep sequencing (Fig 2A). dsDNA cleavage was then detected by examining the frequency of adapter ligation at each position of the protospacer targets. Sequence reads associated with spikes in the frequency of adapter ligation were then examined for biases in the PAM library and, if identified, used as further evidence of target cleavage (Fig 2B). Asp1, Asp2, and Asp3Cas12l all exhibited cleavage peaks after the 23^rd^ and 24^th^ positions of the T2 target region and corresponding library fragments showed a preference for a 5' C‐rich motif (Fig 2B, Appendix Fig S1).

Genes encoding Cas1, Cas2, and Cas4 were next removed from Asp2 and Asp3 CRISPR‐Cas12l systems and dsDNA cleavage was reassessed to confirm that only a single protein was required for the observed activity (Appendix Fig S2). As shown in Appendix Fig S2, cleavage activity was lost entirely. Reasoning that the noncoding sequence between the *cas12l* nuclease and *cas1* gene may encode a tracrRNA, it was added back to the Asp2Cas12l locus and dsDNA cleavage activity was restored (Appendix Fig S2). This allowed us to conclude that Cas1, Cas2, and Cas4 are not involved in dsDNA target recognition and cleavage. Furthermore, it demonstrated that the Cas12l nuclease, a noncoding region upstream of the *cas1* gene, and CRISPR array are essential components for the observed dsDNA target cleavage activity.

### Cas12l guide RNAs

**Figure 3:**
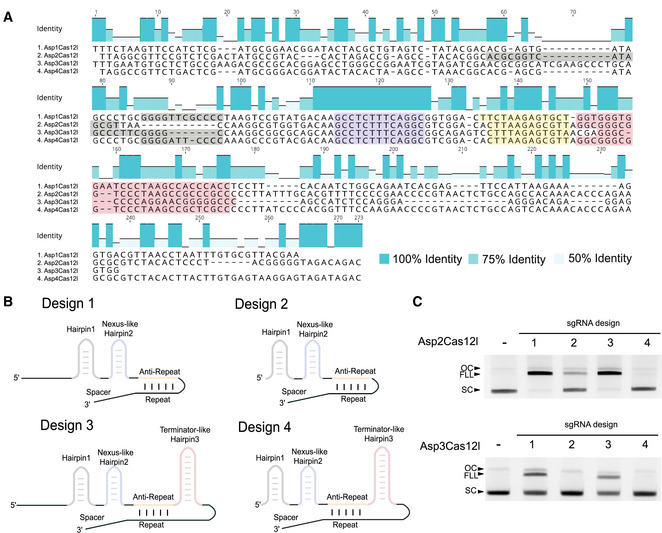
Identification and confirmation of Cas12l guide RNA(s) Alignment of the sequences between the genes encoding the Cas12l effector and Cas1 exhibits features of a trans‐activating CRISPR RNA (tracrRNA). Percent identity is shown in teal. These include a sequence encoding a 5′ hairpin (Hairpin 1—gray), a conserved sequence encoding a nexus‐like stem‐loop (Hairpin 2—blue), a region capable of base pairing with the CRISPR repeat (Anti‐repeat—orange), and a GC‐rich sequence capable of forming a terminator‐like hairpin (Hairpin 3—red).Four single‐guide RNA (sgRNA) designs were engineered for Asp2Cas12l and Asp3Cas12l based on the secondary structure prediction of tracrRNAs in (A). They differed by the presence or omission of a 5′ region of the tracrRNA predicted to be unstructured and by the inclusion or exclusion of the terminator‐like Hairpin 3. tracrRNA features are colored as described in (A).Cleavage of supercoiled (SC) plasmid DNA substrates using purified Cas12l nuclease and respective sgRNA variants. Efficient full‐length linearization (FLL) of the substrate resulting from a complete double‐strand break was only observed when using sgRNAs bearing the 5′ most end of the tracrRNA (variants 1 and 3). Moreover, the terminator‐like Hairpin 3 is not required for target cleavage (variant 1). OC, open circular; FLL, full‐length linear; SC, supercoiled. Source data are available online for this figure.

The guide RNA responsible for directing Cas12l effectors was next determined. Based on the outcome of the CRISPR locus deletion experiments, the noncoding region between the nuclease and *cas1* genes was first searched for the presence of a tracrRNA. Here, a 12–13 bp region with complementation to the CRISPR repeat, an anti‐repeat, was identified for Asp1, Asp2, Asp3, and Asp4Cas12l (Fig 3A). Sequence alignments and secondary structures predictions were then used to define regions of similarity. At the sequence level, Asp1, Asp2, Asp3, and Asp4Cas12l were observed to contain a 17 bp region of 100% similarity immediately 5′ of the anti‐repeat (Fig 3A). Secondary structure analysis showed this region, when transcribed as an RNA, to form a stem‐loop structure reminiscent of a nexus‐like hairpin (Fig 3A and B) observed in the guide RNA of other Cas9 and Cas12 systems (Briner *et al*, 2014; Faure *et al*, 2018; Dooley *et al*, 2021) (Fig 3A and B). Analysis of other RNA structures revealed additional conservation among all four systems. This included Hairpins 1 and 3 located 5′ of the nexus‐like stem loop and 3′ of the anti‐repeat, respectively (Fig 3A and B).

To confirm these sequences as bona fide tracrRNAs, sgRNAs were designed by linking the 3′ end of the putative tracrRNAs with the 5′ end of the CRISPR repeat with a tetraloop, 5′‐GAAA‐3′. Then, using purified Asp2 and Asp3Cas12l nucleases was tested *in vitro* for the ability to guide dsDNA cleavage. Based on the respective putative tracrRNA (Fig 3A), four sgRNA design variants were generated for each effector protein. The sgRNAs differed in length at the 5′ end of the tracrRNA predicted to be unstructured and by the presence or omission of the terminator‐like hairpin (Fig 3B). Only the sgRNA variants bearing the 5′ untruncated portion of the tracrRNA permitted dsDNA cleavage and inclusion of Hairpin 3 reduced cleavage efficiency (Fig 3C). To optimize further, we incrementally truncated the 5′ ends and modified various positions, which formed bulges or mismatches to increase the stability, according to secondary structure predictions, for the Asp2Cas12l‐sgRNA (Appendix Fig S3A). Nevertheless, neither strategy yielded cleavage efficiency superior to the initial full‐length Design 1 sgRNA molecule (Appendix Fig S3B). Finally, sgRNA spacer length was varied between 18 and 24 nts and the effect on target cleavage efficiency evaluated. Both Asp2 and Asp3Cas12l preferred a 20 nt spacer length as averaged across three sites (Appendix Fig S3C). Therefore, Design 1 sgRNAs with a 20 nt length spacer were used in all subsequent experiments.

### Cas12l dsDNA target cleavage

**Figure 4:**
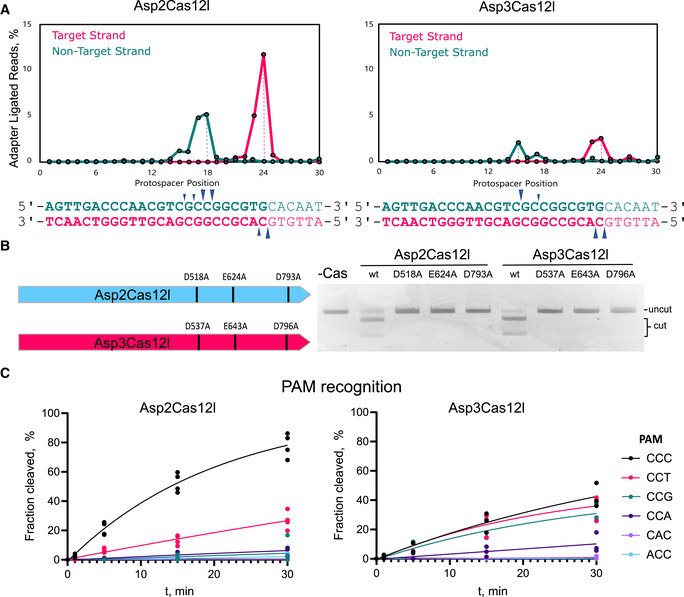
Biochemical characterization of Cas12l dsDNA cleavage Positions of Asp2Cas12l and Asp3Cas12l target and nontarget strand cleavage. Frequency of adater‐ligated reads from PAM library cleavage experiments for both target and nontarget strands. The target strand is cleaved 23–24 nt 3′ of the PAM and the nontarget strand is cleaved 15–18 nt downstream of PAM recognition.Substitution of alanine residues disrupts linear dsDNA cleavage confirming key catalytic positions within the RuvC nuclease domain of Asp2Cas12l and Asp3Cas12l. wt, wildtype.Cleavage of oligoduplex dsDNA substrates with purified RNP complexes confirms PAM recognition. The molar ratio of RNP to the substrate was kept low (5:1) to increase reaction stringency. Asp2Cas12l predominantly recognizes a 5'‐CCY‐3' PAM and Asp3Cas12l a 5'‐CCB‐3' PAM. *N* = 3. Data information: In (C), individual data points are plotted, where *n* = 4 replicates from independent experiments (Asp2Cas12l) and *n* = 3 replicates from independent experiments (Asp3Cas12l). The data points were fitted to a single exponential association curve (solid lines). Source data are available online for this figure.

The positions of the target strand (TS) and nontarget strand (NTS) dsDNA cleavage were evaluated next. Purified Asp2 and Asp3Cas12l ribonucleoproteins (RNPs) were used to digest the 7N plasmid library and cleavage products characterized by adapter ligation, PCR enrichment, and deep sequencing. The position of cleavage within the TS was as observed using *E. coli* lysate above, although most cleavages occurred immediately after the 24^th^ nt downstream of the PAM, and NTS cleavage ensued just after positions 15–18 nts 3′ of the PAM (Fig 4A). To corroborate these results, plasmid DNA containing a fixed nonrandomized PAM and protospacer target was constructed and run‐off Sanger sequencing performed across both ends of the Cas12l cleaved substrate. Although slight variation in the predominant position of TS and NTS cleavage was detected for the two target sequences evaluated, results were consistent with those observed when using the PAM library as substrate (Fig 4A and Appendix Fig S4). Taken together, this shows that Asp2 and Asp3Cas12l proteins cleave dsDNA targets with a 5–9 nt 5′ staggered overhang (Fig 4A).

The RuvC‐like motif identified in Cas12l effectors was next confirmed to be responsible for the observed nuclease activity. This was accomplished by substituting alanine residues in place of the key catalytic D‐E‐D triad within the nuclease‐fold for Asp2 and Asp3Cas12l effectors. Individually, each substitution abolished dsDNA cleavage while retaining target binding, ultimately, confirming structural predictions (Fig 4B and Appendix Fig S5). Cas12l divalent metal ion requirements were examined next. Of those tested (Cu^2+^, Ca^2+^, Ni^2+^, Mn^2+^, Co^2+^, and Mg^2+^), only Mg^2+^ supported dsDNA cleavage (Appendix Fig S6).

### Cas12l PAM recognition

Cas12l PAM recognition was next evaluated using purified components. sgRNAs were first generated for Asp4Cas12l as described for Asp2 and Asp3 and purified Cas12l nucleases complexed with their respective sgRNA. The resulting RNPs were then incubated with the PAM library. Similar to the data generated with *E. coli* lysate, all proteins strongly preferred a C at positions −2 and −3 with Asp2 and Asp3Cas12l also preferring a C at −1 (Appendix Fig S7). To confirm these findings, Asp2 and Asp3Cas12l PAM recognition was interrogated further using substrates with fixed nonrandomized PAM sequences. Here, the replacement of C at positions −2 and −3 completely abolished dsDNA target cleavage for both Asp2 and Asp3Cas12l effectors. At −1, Asp2Cas12l strongly preferred a C and tolerated a T (Fig 4C). By contrast, Asp3Cas12l was observed to be less stringent in accepting C, T, and G and showed weak activity with an A at this position (Fig 4C).

### Effect of DNA supercoiling on Cas12l target cleavage efficiency

**Figure EV1:**
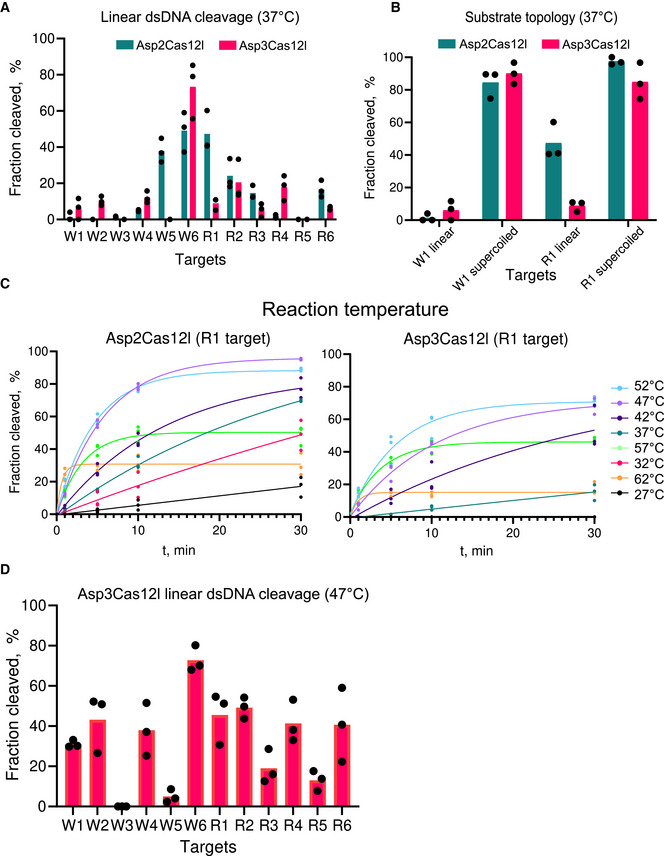
Effect of substrate topology and temperature on Cas12l dsDNA cleavage Cas12l dsDNA hydrolysis efficiency varies depending on the target sequence.Linear and supercoiled dsDNA substrates with different protospacer sequences (W1 and R1) were interrogated by Asp2Cas12l and Asp3Cas12l RNPs. With a linear topology, only the R1 protospacer target was appreciably cleaved; however, both protospacers were cleaved with similar efficiencies by both proteins when presented in a supercoiled state.Effect of reaction temperature on Asp2Cas12l and Asp3Cas12l dsDNA hydrolysis. Optimal temperature for dsDNA cleavage by Asp2Cas12l and Asp3Cas12l RNP complexes is ~50°C.Asp3Cas12l dsDNA hydrolysis across various targets at the increased reaction temperature. Data information: In (A, B, and D), data are presented as mean with individual data points plotted, where *n* = 3 replicates from independent experiments. In (C), individual data points from *n* = 3 replicates from independent experiments are plotted and fitted to a single exponential association curve (solid lines). Source data are available online for this figure.

Next, variation in Cas12l dsDNA target cleavage efficiency was assessed. For this, 12 targets were selected from two therapeutically relevant human genes, WTAP and RunXI, DNA regions PCR‐amplified, and cleavage efficiency evaluated *in vitro* at 37°C using Asp2 and Asp3Cas12l nucleases. Interestingly, many of the targets were not efficiently cleaved (Fig EV1A). Since most of the previous biochemical experimentation used plasmid DNA targets, we reasoned that Cas12l nucleases may prefer supercoiled substrates. To test this, the efficiency of dsDNA target cleavage at the R1 and W1 sites was assayed in both linear and supercoiled states. Asp2 and Asp3Cas12l rapidly cleaved both sites when presented in a supercoiled form (Fig EV1B). By contrast, only the R1 target was partially cleaved by Asp2Cas12l when linear substrates were used (Fig EV1B).

**Figure 5:**
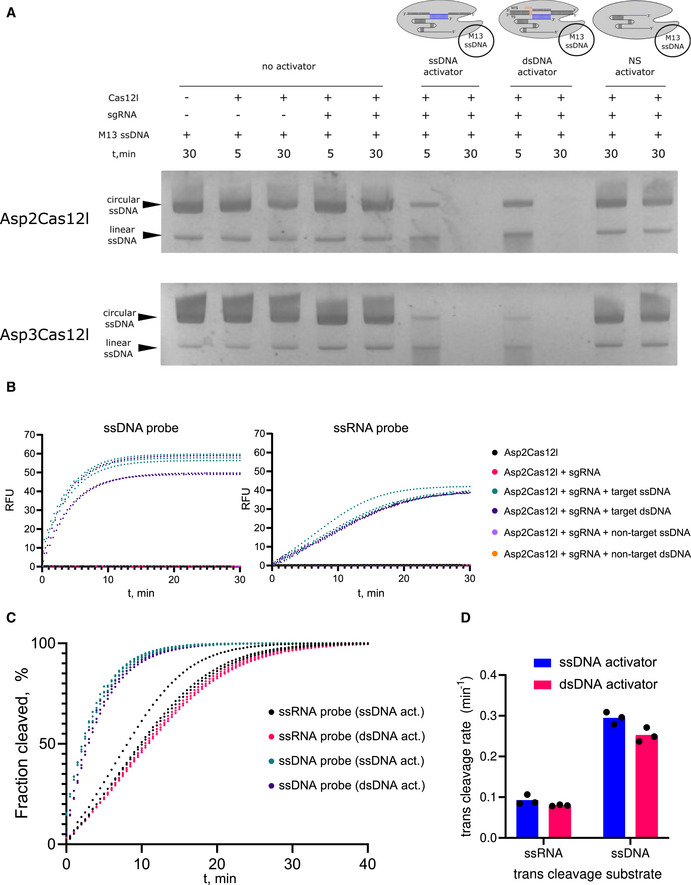
Cas12l collateral nuclease activity Asp2 and Asp3Cas12l RNP complexes degrade M13 ssDNA in the presence of single‐stranded (ss) DNA or dsDNA (including PAM) activators with target sequences complementary to the gRNA spacer. NS activator—nonspecific activator (ssDNA oligonucleotide or dsDNA duplex) with no sequence complementarity to the spacer of the gRNA.Asp2Cas12l RNP complexes activated with ss or dsDNA degrade quenched fluorescent ssDNA or ssRNA probes. Background‐subtracted traces from fluorescent reporter assays with favored ssDNA (5'‐CCCCCCCC‐3′) or ssRNA (5'‐CCCCCCCC‐3′) probes. RFU, relative fluorescence units.Background‐subtracted traces of fluorescent reporter cleavage using ssDNA or ssRNA probes.Rates of trans‐degradation with ssRNA or ssDNA reporters activated with either ssDNA or dsDNA targets. Collateral ssDNAse activity is about 3‐fold higher than the rate of ssRNA degradation. Fluorescence intensities in (B and C) were normalized against the fluorescence of a reaction containing only the probe to account for imperfect quenching or degradation of reporters. Data information: In (B–D), individual data points from *n* = 3 replicates from independent experiments are plotted. In (D), bars represent the mean. Source data are available online for this figure.

**Figure EV2:**
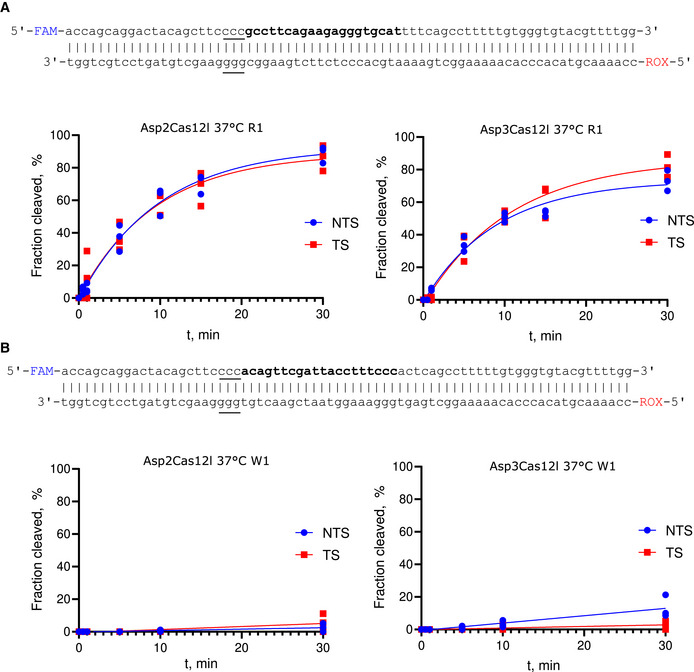
Asp2Cas12l and Asp3Cas12l cleave both dsDNA strands at similar rates *in vitro* Fluorescently labeled (5′‐6‐FAM depicted in blue and 5′‐6‐ROX in red) linear oligoduplex dsDNA substrate with 5'‐CCC‐3' PAM (underlined) and R1 protospacer sequence (bold) used for hydrolysis experiments and cleavage rates of nontarget (NTS) and target (TS) DNA strands.Fluorescently labeled (5′‐6‐FAM depicted in blue and 5′‐6‐ROX in red) linear oligoduplex dsDNA substrate with 5'‐CCC‐3' PAM (underlined) and W1 protospacer sequence (bold) used for hydrolysis experiments and cleavage rates of nontarget (NTS) and target (TS) DNA strands. Data information: Individual data points from *n* = 3 replicates from independent experiments are plotted and fitted to a single exponential association curve (solid lines). Source data are available online for this figure.

Since dsDNA nicking products due to incomplete target cleavage cannot be resolved with linear dsDNA substrate (Fig EV1A), fluorescently labeled oligoduplexes containing R1 and W1 targets were interrogated with both nuclease active and inactive Asp2 and Asp3Cas12l RNP complexes. Nuclease active RNPs were used to assay rates of NTS and TS cleavage. As shown in Fig EV2, Asp2 and Asp3Cas12l RNPs cleaved NTS and TS at similar rates. To probe whether low DNA cleavage efficiency could result because of poor target DNA binding, electrophoretic mobility shift assays were next performed (Appendix Fig S5). This revealed that reduced Asp2 and Asp3Cas12l dRNP binding efficiency at the W1 site indeed correlated with low cleavage efficiency.

Next, the effect of temperature on dsDNA target cleavage was evaluated. For these experiments, linear 900 bp dsDNA fragments containing the R1 target site were used as a substrate. As shown in Fig EV1C, both Asp2 and Asp3Cas12l RNP complexes functioned optimally at elevated temperatures, around 50°C, with Asp2Cas12l demonstrating activity under a wider range of temperatures (Fig EV1C). After 30 min., it was able to cleave over 40% of its substrate at all temperatures except 27°C while Asp3Cas12l activity dropped off precipitously at temperatures below 42°C (Fig EV1C). At temperatures above 52°C, activity diminished for both enzymes (Fig EV1C). Based on these observations, the activity of Asp2 and Asp3Cas12l at the W1 site was re‐evaluated at 47°C. At this temperature, Asp3Cas12l efficiently cleaved the linear substrate, but Asp2Cas12l did not (Appendix Fig S8). This trend continued for Asp3Cas12l across the larger collection of sites where it continued to show better rates of linear dsDNA target cleavage at 47°C (Fig EV1D).

### Cas12l collateral nucleic acid activity

A feature shared by most Type V Cas effectors is the nonspecific collateral degradation of ssDNA after DNA target recognition (Chen *et al*, 2018; Yan *et al*, 2019). To determine whether Cas12l nucleases have this attribute, Asp2 and Asp3Cas12l RNPs were incubated in the presence or absence of an ssDNA or dsDNA target and bacteriophage M13 ssDNA. As shown in Fig 5A, both target substrates triggered the rapid loss of the M13 ssDNA resulting in its near‐complete degradation after 30 min. Also, when both RNPs were incubated with an ss or dsDNA molecule with no sequence complementary to the guide RNA, a nonspecific (NS) activator, M13 ssDNA remained intact confirming that the ssDNase‐like activity is only stimulated by the presence of a target DNA (Fig 5A).

ssDNA sequences separated by a quencher moiety and fluorophore were next used to measure rates of *trans*‐degradation for Asp2Cas12l as described earlier using Cas12a (Chen *et al*, 2018). The effect of sequence length and context of single‐strand nucleic acid probes were first evaluated. Here, it was found that short single‐stranded DNA sequences (8 nts) comprised of cytosine, thymine, or a mix of thymine and adenine residues produced the highest fluorescence relative to the background (Appendix Fig S9A). As reported with Cas12a (Fuchs *et al*, 2022), we also observed that Asp2Cas12l nonspecifically degraded ssRNA molecules and, from those tested, a cytosine‐rich probe resulted in the most rapid accumulation of fluorescence over background (Appendix Fig S9B). Next, collateral nuclease activity, using the favored ssDNA and ssRNA probes, was confirmed to be triggered only in the presence of an ssDNA or dsDNA target, and the rate of ssDNA and ssRNA degradation calculated (Fig 5B–D). Altogether, it was found that ssRNA was nonspecifically cleaved about 3 times slower than ssDNA (Fig 5D) similar to that observed earlier with Cas12a (Fuchs *et al*, 2022).

**Figure EV3:**
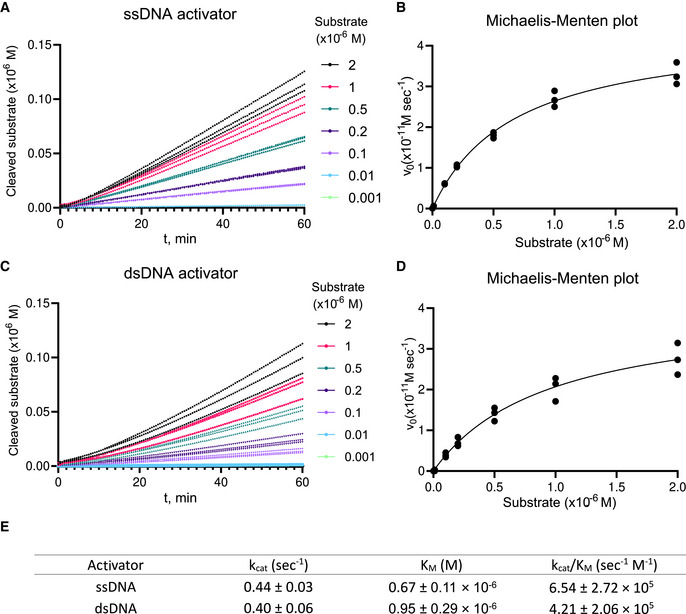
Michaelis–Menten analysis of Asp2Cas12l collateral ssDNA cleavage activity Background‐subtracted traces and corresponding linear trendlines of cleaved substrate concentration versus time for an ssDNA activator, using 0.1 nM effective Asp2Cas12l‐sgRNA‐activator complex and increasing ssDNA reporter concentration.Michaelis–Menten fits for the ssDNA activator.Background‐subtracted traces and corresponding linear trendlines of cleaved substrate concentration versus time for a dsDNA activator, using 0.1 nM effective Asp2Cas12l‐sgRNA‐activator complex and increasing ssDNA reporter concentration.Michaelis–Menten fits for the dsDNA activator.Calculated kinetic constant values. Data information: Individual data points from *n* = 3 replicates from independent experiments are plotted. In (E), data are presented as mean ± SD. See Materials and Methods for the procedure of analysis. Source data are available online for this figure.

To allow the trans‐degradation activity of Asp2Cas12l to be compared with LbaCas12a, the kinetics of ssDNA collateral cleavage by Asp2Cas12l were next examined. For this, the rate of ssDNA collateral degradation was measured using a fixed concentration of Asp2Cas12l‐sgRNA or LbaCas12a‐crRNA RNP complex, 0.1 nM, activated with either an ssDNA or dsDNA target at different ssDNA reporter concentrations, 0.001 × 10^−6^ to 2 × 10^−6^ M, at 37°C. Collateral nuclease activity was measured by fluorescence continuously for 60 min. Raw fluorescence values were converted to cleaved substrate concentrations using standard curves based on the data from experiments assembled without RNP complex and ones allowed to proceed to completion (Fig EV3A and C, Appendix Figs S10, and S11A and C). Michaelis–Menten plots were then fitted to the data and rate of Asp2Cas12l collateral nuclease activity under substrate saturating (k_cat_) and limiting conditions (k_cat_/K_M_) calculated as well as the reporter concentration that provided half‐maximal velocity (K_M_) (Fig EV3B, D, and E). Altogether, it was shown to nonspecifically degrade ssDNA at a rate of 0.44 and 0.41 molecules per second with a k_cat_/K_m_ catalytic efficiency of ~6.5 × 10^5^ or 4.2 × 10^5^ s^−1^ M^−1^ when using an ssDNA or dsDNA activator, respectively (Fig EV3E). Under the same conditions, LbCas12a was shown to nonspecifically degrade ssDNA at a rate of 1.47 or 1.33 molecules per second with a k_cat_/K_M_ catalytic efficiency of 3.9 × 10^7^ or 2.6 × 10^7^ s^−1^ M^−1^ when using an ssDNA or dsDNA activator, respectively (Appendix Fig S11B, D and E).

**Figure 6:**
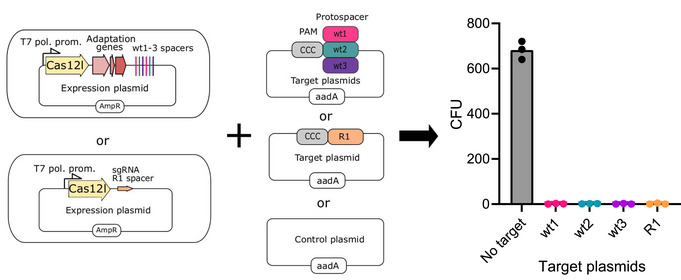
Asp2Cas12l functions in a heterologous cell to protect against invading dsDNA *Escherichia coli* was co‐transformed with plasmids encoding an inducible Asp2Cas12l gene and guide RNA (expression plasmid) and a second plasmid containing the protospacer target(s) adjacent to a suitable PAM for Asp2Cas12l (5'‐CCC‐3′) (target plasmid) or a control plasmid with no PAM or protospacer target sequence. The transformants were plated on a medium containing T7 expression inductor (IPTG) and appropriate antibiotics (carbenicillin and streptomycin). Colonies were only recovered when the control plasmid lacking a target sequence was used. AmpR, ampicillin (carbenicillin) resistance gene, aadA, streptomycin resistance gene. Data information: Data are presented as mean with individual data points from *n* = 3 replicates from independent experiments plotted.

### Defense against invading dsDNA

Since Asp2Cas12l was more active at 37°C than Asp3Cas12l (Fig EV1C), it was next tested for its ability to interfere with DNA plasmid transformation in *E. coli*. Asp2Cas12l effector and guide RNA were expressed from a plasmid containing an ampicillin resistance marker (*AmpR*) in two configurations. The first contained the Asp2Cas12l CRISPR locus while the second encoded only the *Asp2Cas12l* nuclease gene and sgRNA (Fig 6). In both cases, guide RNAs encoded in the plasmid (either CRISPR array or sgRNA) were engineered to target a second plasmid containing a streptomycin resistance (*aadA*) gene (Fig 6). *E. coli* cells were then co‐transformed with the respective AmpR and aadA plasmids and plated on media containing ampicillin and streptomycin. Only in the case of a control plasmid, lacking a target sequence, were any colonies observed (Fig 6).

## Discussion

In this study, a new family of type V CRISPR nuclease, designated here as Cas12l, is identified and characterized. From a relatively new phylum of bacteria, *Armatimonadetes* previously titled OP10 (Tamaki *et al*, 2011), these new type V systems further potentiate that the natural diversity afforded by CRISPR‐Cas systems continues to provide a rich source for the discovery and development of novel RNA‐guided nucleases. Ranging in size from 854–867 aa, they are smaller than most Cas12 effector nucleases only surpassed in compactness by type V‐J (700–800 aa) and V‐F (400–700 aa) systems (Pausch *et al*, 2020; Bigelyte *et al*, 2021). Moreover, the collection of Cas12l nucleases identified here is exclusively triggered to cleave dsDNA in the presence of a 5' C‐rich PAM. While CRISPR‐Cas9 nucleases have been shown to recognize a C‐rich PAM (Edraki *et al*, 2019; Gasiunas *et al*, 2020), this feature in a Cas12 effector makes them a desirable addition to the type V family providing an important counterbalance to the 5′ T‐rich PAM recognition typified by most other Cas12 enzymes for biotechnology and nucleic acid detection applications. Their guide RNA is comprised of a tracrRNA and crRNA and can be engineered as a sgRNA like other type V family members (Shmakov *et al*, 2015; Burstein *et al*, 2017; Yan *et al*, 2019). In our experimentation, both Asp2Cas12l and Asp3Cas12l prefer supercoiled substrates over linear ones and lack of target binding correlates with the inability to cleave certain linear substrates. This indicates that the unwinding of supercoiled DNA during R‐loop formation helps to facilitate target recognition although this effect was also achieved using incubations at higher temperatures for Asp3Cas12l. Indeed, substrate topology has been shown to be a factor in consistent dsDNA cleavage with supercoiled targets being preferred over relaxed ones for other CRISPR effector complexes including type I‐E, Cas12a, and Cas9 (Westra *et al*, 2012; Van Aelst *et al*, 2019). Taken together, this suggests that negative DNA supercoiling employed by mesophiles to facilitate strand‐opening for many DNA‐dependent processes including transcription factor binding, transcription, and replication (López‐García, 1999; Bates & Maxwell, 2005) are also used by organisms harboring Cas12l CRISPR systems. Additionally, similar to that observed with Cas12a (Chen *et al*, 2018; Fuchs *et al*, 2022), ssDNA and dsDNA target recognition triggers the trans‐degradation of ssDNA and ssRNA. Here, under the same experimental parameters, the turnover of the substrate by Asp2Cas12l is about 3‐fold lower than LbCas12a. This may in part be explained by the observation that Asp2Cas12l nucleolytic activity is more potent at temperatures around 50°C (Fig 5C) while 37°C, which is optimal for LbaCas12a, is utilized in the comparison. Finally, at least one member of the Cas12l family, Asp2Cas12l, mediates protection against invading plasmid DNA in a heterologous *E. coli* host. Taken together, this suggests that this new family of effector nucleases may also be harnessed for use in other cell types.

## Materials and Methods

### Identification of CRISPR‐Cas12l

First, arrays of CRISPRs were detected within microbial sequences using PILER‐CR (Edgar, 2007) and MinCED (Bland *et al*, 2007) software programs. Next, known CRISPR‐Cas systems were removed from the dataset by searching the proteins encoded in the vicinity (20 kb 5' and 20 kb 3′ (where possible)) of the CRISPR array for homology with known CRISPR‐associated (Cas) proteins utilizing a set of position specific scoring matrices (PSSMs) encompassing all known Cas protein families as described in Makarova *et al* (2015). To aid in the complete removal of known Class 2 CRISPR‐Cas systems, multiple‐sequence alignment of protein sequences from a collection of orthologs from each family of Class 2 CRISPR‐Cas endonucleases (e.g., Cas9, Cpf1 (Cas12a), C2c1 (Cas12b), C2c2 (Cas13), C2c3 (Cas12c)) was performed using MUSCLE (Edgar, 2004). The alignments were examined, curated, and used to build profile hidden Markov models (HMM) using HMMER (Eddy, 1998; Finn *et al*, 2011). The resulting HMM models were then utilized to further identify and remove known Class 2 CRISPR‐Cas systems from the dataset. Next, using PSSM‐specific searches as described above, the CRISPR loci that remained were evaluated for the presence of genes encoding proteins implicated as being important for spacer insertion and adaptation, Cas1 and Cas2. CRISPR loci containing *cas1* and *cas2* genes were then selected and further examined to determine the proximity, order, and directionality of the undefined genes encoded in the locus relative to the *cas1* and *cas2* genes and CRISPR array. Only those CRISPR loci forming an operon‐like structure where a large (≥ 1,500 bp open‐reading frame) undefined gene was present close to and in the same transcriptional direction as the *cas1* and *cas2* genes were selected for further analysis. Next, the protein encoded by the undefined gene was analyzed for sequence and structural features indicative of a type V nuclease domain. First, depending on how much similarities existed between a candidate sequence and known proteins, various bioinformatics tools were employed to reveal its conserved functional features, from pairwise comparison to family‐profile search, to structural threading, and to manually structural inspection. In general, homologous sequences for candidate proteins were first collected by a PSI‐BLAST (Altschul *et al*, 1997) search against the National Center for Biotechnology Information (NCBI) nonredundant (NR) protein collection with a cut‐off e‐value of 0.01. After redundancy reduction at ~90% identical level, groups of homologous sequences with various member inclusion thresholds (such as > 60, 40, or 20% identity) were aligned to reveal conserved motifs by multiple‐sequence alignment tools, MSAPRobs (Liu *et al*, 2010) and ClustalW (Thompson *et al*, 1994). The most conserved homologous sequences underwent a sequence to family‐profile search by HMMER (Eddy, 1998), against numerous domain databases including Pfam, Superfamily, and, SCOP (Murzin *et al*, 1995) and home‐built structure‐based profiles. Separately, the resulting candidate's homologous sequence alignment was also used to generate a candidate protein profile with the addition of predicted secondary structures. The candidate profile was further used to do a profile‐profile search by HHSEARCH (Söding, 2005), against pdb70_hhm and Pfam_hhm profile databases. In the next step, all detected sequence‐structure relationships and conserved RuvC‐like motifs were threaded into a 3D structure template with modeler or manually mapped into the known structural reference on DiscoveryStudio (BIOVIA) and Pymol (Schrodinger). Finally, to verify and confirm the potential biological relevance, the catalytic or most conserved residues and key structural integrity were manually inspected and evaluated in light of the protein's ability to metabolize DNA. Following RuvC identification, the other proteins encoded within the locus (5 kb 5′ and 5 kb 3′ from the ends of the newly defined CRISPR‐Cas system (where possible)) were next examined for homology to known proteins families using InterProScan software (EMBL‐EBI, UK) and through comparison with the NCBI NR protein collection using the BLAST program (Altschul *et al*, 1990).

### Engineering CRISPR‐Cas12l systems to target a PAM library

Cas12l CRISPR systems were modified to target the 7 bp randomized PAM library described previously (Karvelis *et al*, 2018). The native CRISPR array was replaced with four repeat:spacer:repeat units, two of which encoded 37 nt spacers capable of base pairing with the anti‐sense strand 5′ of the randomized PAM sequence in the library, and two that encoded sequence capable of targeting the anti‐sense strand 3′ of the randomized PAM region. The resulting engineered CRISPR‐Cas loci were synthesized (GenScript) and cloned into a low copy number *E. coli* plasmid (pETDuet‐1) modified to contain a single isopropyl β‐D‐1‐thiogalactopyranoside (IPTG) inducible T7 promoter (MilliporeSigma). The links to plasmid sequences are listed in the Dataset EV1.

### Detecting Cas12l dsDNA cleavage and PAM recognition

*Escherichia coli* ArcticExpress (DE3; Agilent Technologies) cells were transformed with the plasmid‐borne engineered CRISPR‐Cas12l systems. Cultures were grown in 20 ml of LB media containing 20 μg ml^−1^ gentamycin and 100 μg ml^−1^ carbenicillin. After the cultures reached OD_600_ of 0.6, expression was induced with 0.1 mM IPTG, and the cultures were then incubated overnight at 16°C. Cells from aliquots of 10 ml were collected by centrifugation and resuspended in 1 ml of lysis buffer (20 mM Tris–HCl, pH 7.5, 500 mM NaCl, 5% (v/v) glycerol) supplemented with 1 mM PMSF and lysed by sonication. Cell debris was removed by centrifugation, and 10 μl of the obtained supernatant was used in the plasmid cleavage reactions.

Lysate containing Cas12l RNP complexes was then used to cleave the 7 bp randomized PAM plasmid library. Ten microliter of clarified *E. coli* lysate was mixed with 500 ng of PAM library in 50 μl of reaction buffer (1× CutSmart buffer (New England Biolabs): 50 mM KOAc, 20 mM Tris‐OAc, 10 mM Mg(OAc)_2_, 100 μg ml^−1^ BSA, pH 7.9) and incubated at 37°C for 1 h. Afterward, DNA ends were repaired by adding 0.3 μl of T4 DNA polymerase (New England Biolabs) and 0.3 μl of 10 mM dNTP mix (Thermo Fisher Scientific) and incubating the reaction for 15 min at 12°C. The reaction was then inactivated by heating it up to 75°C for 25 min. To ensure efficient adapter ligation 3′‐dA overhangs were added to the cleavage products by incubating the reaction with 0.3 μl of DreamTaq polymerase (Thermo Fisher Scientific) and 0.3 μl of 10 mM dNTP mix for 30 min at 72°C. RNA was then removed by adding 0.5 μl of RNase A (Thermo Fisher Scientific) and incubating for 15 min at 37°C. The end‐repaired cleavage products were purified with Monarch PCR & DNA Cleanup columns (New England Biolabs) and 100 ng of obtained DNA were ligated with 100 ng of a double‐stranded DNA adapter containing a 3′‐dT overhang for 1 h at 22°C using T4 DNA ligase (New England Biolabs). Ligated cleavage products were then subject to Illumina sequencing and analyzed for evidence of dsDNA target cleavage and PAM recognition as described earlier (Karvelis *et al*, 2018). Briefly, two rounds of PCR were performed adding on the sequences required for Illumina sequencing and subsequent deconvolution using primers with 5′ extensions. Amplifications were then pooled and single‐read sequenced on a MiSeq System (Illumina). Sequence reads were next quality trimmed (Q13) and amplicons separated based on a 5′ 6 nt index incorporated during PCR. The percentage of adapter ligation to each position of the PAM library protospacer target was next calculated as described previously (Karvelis *et al*, 2018). Sequence reads associated with an elevated frequency of adapter ligation were then examined for biases in the randomized PAM region relative to their distribution in the starting library.

### Expression and purification of Cas12l proteins

Cas12l proteins were expressed in *E. coli* NiCo21(DE3) strain (New England Biolabs) from pET‐MBP14xHisSUMO‐Cas12l plasmids. Cells were grown in LB media at 30°C. After the cultures reached an OD_600_ of 0.5, expression was induced with 0.4 mM IPTG and incubation continued at 16°C overnight. Cells were pelleted by centrifugation and resuspended in loading buffer (20 mM Tris–HCl, pH 7.5, 300 mM NaCl, 40 mM imidazole) and subjected to disruption by sonication. Cell debris was removed by centrifugation and supernatant filtered before loading onto a HiTrap DEAE FF chromatography column (GE Healthcare). The flowthrough was then loaded onto a Ni^2+^‐charged HisTrap column (GE Healthcare) and eluted with a linear gradient of increasing imidazole concentration (from 40 to 700 mM). The fractions containing Cas12l proteins were pooled, and the MBP‐14xHis‐SUMO tags were cleaved by increasing the NaCl concentration to 500 mM, adding 1 mM DTT and 2% (v/v) glycerol and 100 nM SenP1 protease and incubating at 4°C overnight. To remove the cleaved tag and SenP1 protease the reaction mixture was loaded onto a HiTrap Heparin column (GE Healthcare) and eluted with a linear gradient of increasing NaCl concentration (from 0.5 to 2 M). Fractions containing Cas12l proteins were pooled and dialyzed against 20 mM Tris–HCl, pH 7.5, 500 mM NaCl, 1 mM DTT, 0.1 mM EDTA, 50% glycerol storage buffer, and stored at −20°C. The protein sequences and links to expression plasmids are listed in the Dataset EV1.

### RNA synthesis

Cas12l single guide RNAs (sgRNA) were produced by *in vitro* transcription using HiScribe T7 Quick High Yield RNA Synthesis Kit (New England Biolabs) and purified using Monarch RNA Cleanup Kit (New England Biolabs). Templates for T7 transcription were generated by PCR amplification of synthesized geneblocks (GenScript). Sequences of the geneblocks and sgRNAs used are provided in the Dataset EV1.

### Cas12l‐sgRNA complex assembly for *in vitro* cleavage

Cas12l‐sgRNA RNP complexes were assembled by mixing purified Cas12l protein with sgRNA at 1:2 molar ratio followed by incubation in a complex assembly buffer (10 mM Tris–HCl, pH 7.5, 100 mM NaCl, 1 mM DTT, 1 mM EDTA) at room temperature for 20 min.

### DNA substrate generation

Plasmid DNA substrates were generated either by cloning oligoduplexes assembled after annealing complementary oligonucleotides containing PAM and protospacer sequences into pUC19 plasmid over EcoRI (New England Biolabs) and HindIII (New England Biolabs) restriction sites or by cloning PCR products containing PAM and protospacer sequences via blunt end ligation over end repaired EcoRI and HindIII restriction sites.

Fluorescently labeled DNA substrates were generated by annealing partially complementary oligonucleotides containing PAM and protospacer sequences and PCR amplifying them with primers containing 5′‐6‐FAM (nontarget strand) or 5′‐6‐ROX (target strand) (IDT) dyes.

The sequences of the DNA substrates and links to plasmid sequences are provided in the Dataset EV1.

### DNA substrate cleavage assays

DNA cleavage reactions were initiated by mixing DNA substrates with Cas12l RNP complexes. Plasmid DNA cleavage reactions were carried out at 37°C in 1× CutSmart buffer (New England Biolabs) with a 1:20 substrate:complex molar ratio, unless stated otherwise. Aliquots were removed at timed intervals (30 min if not indicated differently) and mixed with 6× Blue Gel Loading Dye (New England Biolabs). Reaction products were analyzed by agarose gel electrophoresis and GelRed (Biotium) staining. Fractions of cleaved substrate were calculated by densitometric analysis with ImageJ software according to the following equation: $\text{Fraction}\;\text{cleaved},\;\%=\left(\frac{I_{\text{products}}}{\left(I_{\text{products}}+I_{\text{substrate}}\right)}\right)\times 100$, where $I_{\text{products}}$ is the intensity of cleavage product bands, and $I_{\text{substrate}}$ is the intensity of the intact substrate band.

Reactions with fluorescently labeled DNA substrates were carried out at 37 or 47°C in 1× CutSmart buffer with a 1:10 substrate:complex molar ratio or 1:5 substrate:complex molar ratio (suboptimal PAM substrate cleavage screens; 20 mM substrate and 200 or 100 mM Cas12l complex, respectively, unless stated otherwise). Aliquots were removed at timed intervals and mixed with 9.5 μl Hi‐Di Formamide (Thermo Fisher Scientific) supplemented with 0.5 μl GeneScan LIZ 120 Size Standard (Thermo Fisher Scientific). Reaction products were subjected to capillary electrophoresis using a 3500 Series Genetic Analyzer (Thermo Fisher Scientific) as per manufacturers' recommendations. Electrophoresis data were analyzed with OSIRIS (NCBI) or Geneious Prime (Biomatters Ltd.) software. Fragment size was evaluated by comparing the retention time with Internal Lane Standard retention time and the fractions of cleaved substrate calculated by comparing the substrate and cleavage product fragment peak area (minimum RFU (relative fluorescence units) for peak detection—50):

$$\text{Cleavage,}\;\%=\left(\frac{\text{peak}\;\;\text{area}\;\left(\text{products}\right)}{\text{peak}\;\text{area}\;\left(\text{substrate}\right)+\text{peak}\;\text{area}\;\left(\text{products}\right)}\right)\times 100$$

Cleavage rate graphs were plotted using GraphPad Prism Software.

### M13 ssDNA cleavage assays

M13 ssDNA cleavage reactions were initiated by mixing M13 ssDNA (New England Biolabs) and ssDNA activator (oligonucleotide) or dsDNA activator (oligonucleotide duplex) with Cas12l RNP complexes at 37°C. Cleavage reactions were carried out in 1× CutSmart buffer. Final reaction mixture contained 5 nM M13 ssDNA, 100 nM ssDNA/dsDNA activator, 100 nM Cas12l‐sgRNA complex (unless stated otherwise). Reactions were stopped by mixing with 6× Blue Gel Loading Dye (New England Biolabs) and products analyzed by agarose gel electrophoresis and GelRed staining. The sequences of the activators are listed in the Dataset EV1.

### Electrophoretic mobility shift assays

dCas12l:sgRNA complexes were assembled by combining Cas12l RuvC active center mutants with sgRNA at a 1:2 molar ratio in 1× CutSmart buffer and incubated at room temperature for 20 min. Substrate binding was carried out by mixing 2 nM of fluorescently labeled DNA oligoduplex in 1× CutSmart buffer with increasing concentrations of dCas12l RNP and incubating at 37°C for 30 min. Ten microliter binding reactions were mixed with 2 μl of 6× Purple DNA loading dye (No SDS; NEB), supplemented with 10 mM Mg^2+^, and loaded onto a 5% nondenaturing PAA gel in 0.5× TBE (Thermo Fisher Scientific) buffer, supplemented with 5 mM Mg^2+^. Electrophoresis was carried out in 0.5× TBE buffer, supplemented with 5 mM Mg^2+^ at 4°C for 3 h.

Concentrations of bound substrate were calculated by evaluating the intensity of bands using ImageJ software and converting the intensities according to the following equation: $c_{\text{bound}}=\frac{I_{\text{bound}}}{\left(I_{\text{bound}}+I_{\text{free}}\right)}\times c_{0}$, where $c_{\text{bound}}$ is the concentration of bound substrate, $I_{\text{bound}}$ is the intensity of the shifted band at a given enzyme concentration, $I_{\text{free}}$ is the intensity of the free substrate band at a given RNP concentration, and $c_{0}$ is the initial substrate concentration (2 nM). The concentrations of bound substrate were then plotted against the concentrations of enzyme RNP and fitted to a nonlinear regression (GraphPad Prism Software) according to the following equation: $Y=0.5\times \left(K_{d}+x+S\right)-0.5\times \sqrt{{\left(K_{d}+x+S\right)}^{2}-4\times x\times S}$, where Y is the concentration of bound substrate, $K_{d}$ is the dissociation constant, x is the concentration of enzyme RNP, and S is the initial concentration of substrate (2 nM).

### Fluorophore quencher‐labeled reporter assays

Asp2Cas12l‐sgRNA complexes were preassembled by incubating 500 nM of Asp2Cas12l with 600 nM sgRNA in 1× 2.1 buffer (NEB) at room temperature for 15 min. The complexes were then diluted and ssDNA/dsDNA activators added to a final concentration of 105 nM Asp2Cas12l: 125 nM sgRNA: 26 nM ssDNA/dsDNA activator and incubated at 50°C for 30 min in 1× 2.1 buffer (NEB). The trans‐cleavage reactions were initiated by adding 50 pmol of ssDNA/ssRNA fluorophore quencher (FQ) reporter substrates with the final concentration being 100 nM Asp2Cas12l: 120 nM sgRNA: 25 nM ssDNA/dsDNA activator: 500 nM FQ substrate in a 100 μl reaction volume in a black 96‐well plate. The reactions were immediately placed in a fluorescence plate reader and incubated at 37°C with fluorescence measurements taken every 30 s.

For trans‐cleavage rate determination, raw fluorescence values were corrected by subtracting fluorescence values obtained from reactions with FQ reporters only. The relative fluorescence values were then converted to FQ substrate fraction cleaved according to the following equation: $\text{Fraction}\;\text{cleaved,}\;\%=\frac{F\left(t\right)}{F\left(\text{cleaved}\right)}\times 100$, where $F\left(t\right)$ is the fluorescence at a given time point; $F\left(\text{cleaved}\right)$ is the fluorescence of fully cleaved FQ reporter. The resulting data were fit to a single exponential decay curve (GraphPad Software), according to the following equation: $\text{Fraction cleaved}=A\times \left(1-e^{\left(-k\times t\right)}\right)$, where *A* is the amplitude of the curve; *k* is the pseudo‐first‐order rate constant and *t* is time.

For Michaelis–Menten analysis, 400 nM Asp2Cas12l: 500 nM sgRNA: 4 nM ssDNA/dsDNA activator complexes were assembled by first incubating Asp2Cas12l and sgRNA in 1× 2.1 buffer (NEB) at room temperature for 15 min and then adding the ssDNA/dsDNA activators and incubating at 50°C for 30 min. The trans‐cleavage reactions were initiated by diluting the effective complexes to final concentrations of 10 nM Asp2Cas12l: 12.5 nM sgRNA: 0.1 nM activator in a solution containing 1× 2.1 buffer and 0.001, 0.01, 0.1, 0.2, 0.5, 1, or 2 μM fluorophore quencher‐labeled substrate in a 100 μl reaction volume in a black 96‐well plate. The reactions were incubated in a fluorescence plate reader at 37°C for up to 60 min with fluorescence measurements taken at every 30 s (λ_ex_ = 485 nm; λ_em_ = 538 nm). Reactions containing no Asp2Cas12l: sgRNA: activator complexes were measured to obtain uncleaved reporter fluorescence versus concentration standard curves and reactions containing a 100‐fold higher concentration of Asp2Cas12l: sgRNA: activator effective complexes were measured to obtain fully cleaved reporter fluorescence versus concentration standard curves (Appendix Fig S10). Reactions with LbaCas12a were carried out under the same conditions, except an additional reaction with the fluorophore quencher substrate concentration of 0.05 μM was used in the analysis.

Relative fluorescence values obtained were converted to cleaved fluorescent substrate concentrations according to the following equation: $c_{\mathrm{cl}}=\frac{F\left(t\right)-F\left(c_{0}\right)}{S_{\mathrm{cl}}-S_{\mathrm{ucl}}}$, where $c_{\mathrm{cl}}$ is the cleaved substrate concentration; $F\left(t\right)$ is the relative fluorescence value at a given time point; $F\left(c_{0}\right)$ is the fluorescence value of uncleaved reporter at a given concentration; $S_{\mathrm{cl}}$ is the slope of a linear standard curve obtained by plotting relative fluorescence values of fully cleaved substrate versus substrate concentration and fitting to linear regression; $S_{\mathrm{ucl}}$ is the slope of a linear standard curve obtained by plotting relative fluorescence values of uncleaved substrate versus substrate concentration and fitting to linear regression (Appendix Fig S10). A fully cleaved substrate was generated by incubating with Asp2Cas12l until the RFU values reached a stable peak.

Initial velocity (v_0_) was calculated by fitting the cleaved substrate concentration versus time traces to linear regression and plotted against the initial substrate concentration. Michaelis–Menten constants were determined according to the following equation: $Y=\frac{V_{\max}\times X}{K_{M}+X}$, where X is the substrate concentration and Y is the enzyme velocity (GraphPad Prism Software). The turnover number (k_cat_) was determined by the following equation: $k_{\mathrm{cat}}=\frac{V_{\max}}{E}$, where E is the effective complex concentration (0.1 nM).

### Plasmid interference assay

Plasmid interference experiments were carried out in *E. coli* Arctic Express (DE3; Agilent Technologies) strain. First, pETDuet plasmid bearing the *Asp2Cas12l* gene was engineered to carry spacers found in native Cas12l loci by cloning a synthesized DNA fragment (containing repeat‐spacer1‐repeat‐spacer2‐repeat‐spacer3‐repeat sequence) over restriction sites. Another pETDuet plasmid containing the *cas12l* gene was constructed by adding a sgRNA coding sequence bearing a RunXI spacer with T7 promoter, HDV ribozyme, and terminator sequences, using the Gibson assembly molecular cloning method (New England Biolabs). Next, pCDF‐Duet plasmids were engineered to carry protospacer sequences, complementary to the native spacers and RunXI spacer found in the pETDuet plasmids, downstream of corresponding PAM 5'‐CCC‐3′ by cloning oligoduplexes over restriction sites in the pCDF‐Duet plasmid.

*Escherichia coli* Arctic Express (DE3; Agilent Technologies) cells were first transformed with pETDuet plasmids containing the *Asp2Cas12l* gene, gRNA, and spacer sequences. These cells were grown at 37°C to an OD600 of 0.5 and electroporated with 200 ng of target pCDF‐Duet plasmids containing protospacer sequences or an empty pCDF‐Duet control plasmid. The co‐transformed cells were grown at 37°C for 16–20 h on plates containing 100 g ml^−1^ carbenicillin, 10 g ml^−1^ streptomycin, 10 g ml^−1^ gentamycin, and 0.1 mM IPTG. Plasmid interference was evaluated by the amount of transformant colonies observed on the plate.

## Author contributions

**Tomas Urbaitis:** Conceptualization; data curation; formal analysis; validation; investigation; visualization; methodology; writing – original draft; writing – review and editing. **Giedrius Gasiunas:** Conceptualization; data curation; formal analysis; supervision; visualization; writing – original draft; writing – review and editing. **Joshua K Young:** Conceptualization; data curation; software; formal analysis; supervision; validation; investigation; visualization; methodology; writing – original draft; writing – review and editing. **Zhenglin Hou:** Software; formal analysis; investigation; methodology; writing – review and editing. **Sushmitha Paulraj:** Investigation; methodology; writing – review and editing. **Egle Godliauskaite:** Investigation; methodology; writing – review and editing. **Mantvyda M Juskeviciene:** Investigation; methodology; writing – review and editing. **Migle Stitilyte:** Investigation; methodology; writing – review and editing. **Monika Jasnauskaite:** Investigation; methodology; writing – review and editing. **Megumu Mabuchi:** Investigation; methodology; writing – review and editing. **G Brett Robb:** Data curation; formal analysis; writing – review and editing. **Virginijus Siksnys:** Formal analysis; writing – review and editing.

## Disclosure and competing interests statement

ZH, JKY, TU, and GG have filed patent applications related to the paper. TU, GG, EG, MMJ, and MS are employees of CasZyme. MJ was an employee of CasZyme (current address: LSC‐EMBL Partnership Institute for Genome technologies Editing, Life Sciences Center, Vilnius university). JKY, ZH, and SP are employees of Corteva Agriscience. MM and GBR are employees of NEB. VS is the Chairman of CasZyme. VS and GG have a financial interest in CasZyme. The remaining authors declare that they have no conflict of interest.

## Supporting information




Appendix

Expanded View Figures PDF

Dataset EV1

Source Data for Figure 3

Source Data for Figure 4

Source Data for Figure 5

Source Data for Expanded View and Appendix

PDF+

## Data Availability

All data can be found in the manuscript or Supplementary Material. All Illumina sequence data produced when assessing the position of Cas12l target cleavage and PAM preferences have been deposited at NCBI under Sequence Read Archive PRJNA865310.
