# Supplementary material for: A new family of CRISPR‐type V nucleases with C‐rich PAM recognition
Source: EMBO Rep. 2022 Oct 21;23(12):e55481. doi: 10.15252/embr.202255481 (PMC9724661; doi:10.15252/embr.202255481)
Supplement: Supplementary file 1 — Appendix [file EMBR-23-e55481-s006.pdf]

Appendix for

A new family of CRISPR-type V nucleases with C-rich PAM recognition

Tomas Urbaitis, Giedrius Gasiunas, Joshua K. Young, Zhenglin Hou, Sushmitha Paulraj, Egle Godliauskaite, Mantvyda M. Grusyte, Migle Stitilyte, Monika Jasnauskaite, Megumu Mabuchi, G. Brett Robb, and Virginijus Siksnys

Table of contents:

Appendix Figure S1 - page 2

Appendix Figure S2 - page 2

Appendix Figure S3 - page 3

Appendix Figure S4 - page 4

Appendix Figure S5 - page 5

Appendix Figure S6 - page 6

Appendix Figure S7 - page 7

Appendix Figure S8 - page 8

Appendix Figure S9 - page 9

Appendix Figure S10 - page 9

Appendix Figure S11 -page 10

A

PAM position

Asp1Cas12l

Nucleotide	-7	-6	-5	-4	-3	-2	-1
T	0.28	0.26	0.26	0.25	0.01	0.00	0.38
G	0.24	0.25	0.23	0.25	0.01	0.01	0.04
C	0.23	0.26	0.22	0.17	0.98	0.98	0.54
A	0.26	0.23	0.29	0.33	0.01	0.01	0.04
	N	N	N	N	C	C	Y

B

PAM position

Asp2Cas12l

Nucleotide	-7	-6	-5	-4	-3	-2	-1
T	0.22	0.25	0.26	0.29	0.00	0.01	0.14
G	0.28	0.31	0.28	0.30	0.00	0.00	0.00
C	0.20	0.23	0.23	0.13	0.99	0.99	0.85
A	0.29	0.22	0.23	0.28	0.00	0.00	0.00
	N	N	N	N	C	C	C

C

PAM position

Asp3Cas12l

Nucleotide	-7	-6	-5	-4	-3	-2	-1
T	0.29	0.31	0.30	0.23	0.00	0.00	0.43
G	0.23	0.20	0.22	0.25	0.00	0.00	0.11
C	0.23	0.27	0.23	0.13	0.99	1.00	0.43
A	0.25	0.22	0.25	0.39	0.00	0.01	0.03
	N	N	N	N	C	C	Y

Appendix Figure S1. Position weight matrices of PAM sequences that supported dsDNA cleavage. (A) Lysate expressing Asp1Cas12l; (B) Lysate expressing Asp2Cas12l; (C) Lysate expressing Asp3Cas12l.

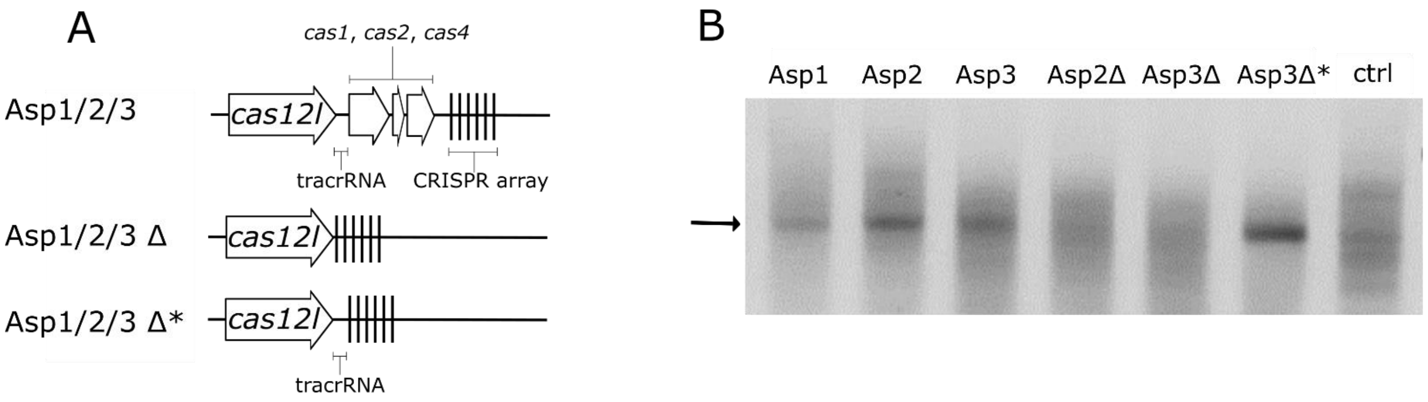

Appendix Figure S2. PAM plasmid library hydrolysis with lysates expressing Cas12l loci deletion variants. (A) Schematics of the loci used in each experiment. Asp1/2/3 - full locus of each respective Cas12l ortholog, Asp1/2/3 Δ - locus with adaptation genes (*cas1*, *cas2* and *cas4*) and putative tracrRNA transcription region removed, Asp1/2/3 Δ* - locus with adaptation genes removed but putative tracrRNA region intact. (B) PCR products from

adapter-captured PAM library cleavage. Ctrl – negative control – lysate of E. coli not transformed with any plasmid.

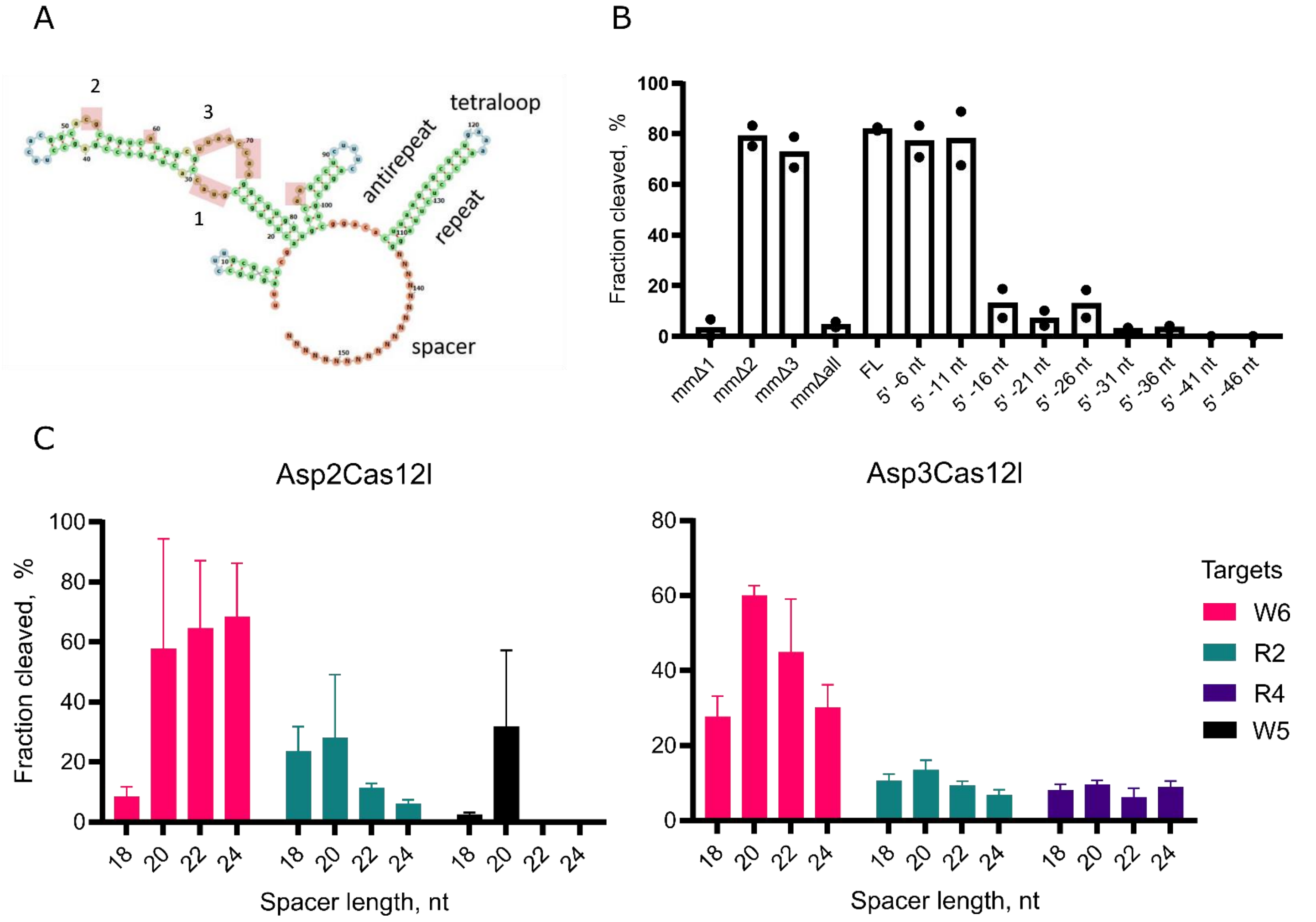

Appendix Figure S3. Cas12l guide RNA optimization. (A) Predicted Asp2Cas12l sgRNA secondary structure. Regions that were removed are highlighted in red and denoted with numbers. (B) Cleavage of oligoduplex dsDNA substrates with Asp2Cas12l protein and respective sgRNA deletion variants. mmΔ1,2,3 - sgRNA with nucleotides removed as depicted in (A), mmΔall - sgRNA with nucleotides highlighted in red in (A) removed, FL - full length sgRNA, 5' -x nt - sgRNA with x nucleotides removed from 5' end. N=2 (C) Effect of sgRNA spacer length on AspCas12l linear dsDNA substrate cleavage. Data in (C) presented as mean ± s.d., where n = 3 replicates from independent experiments.

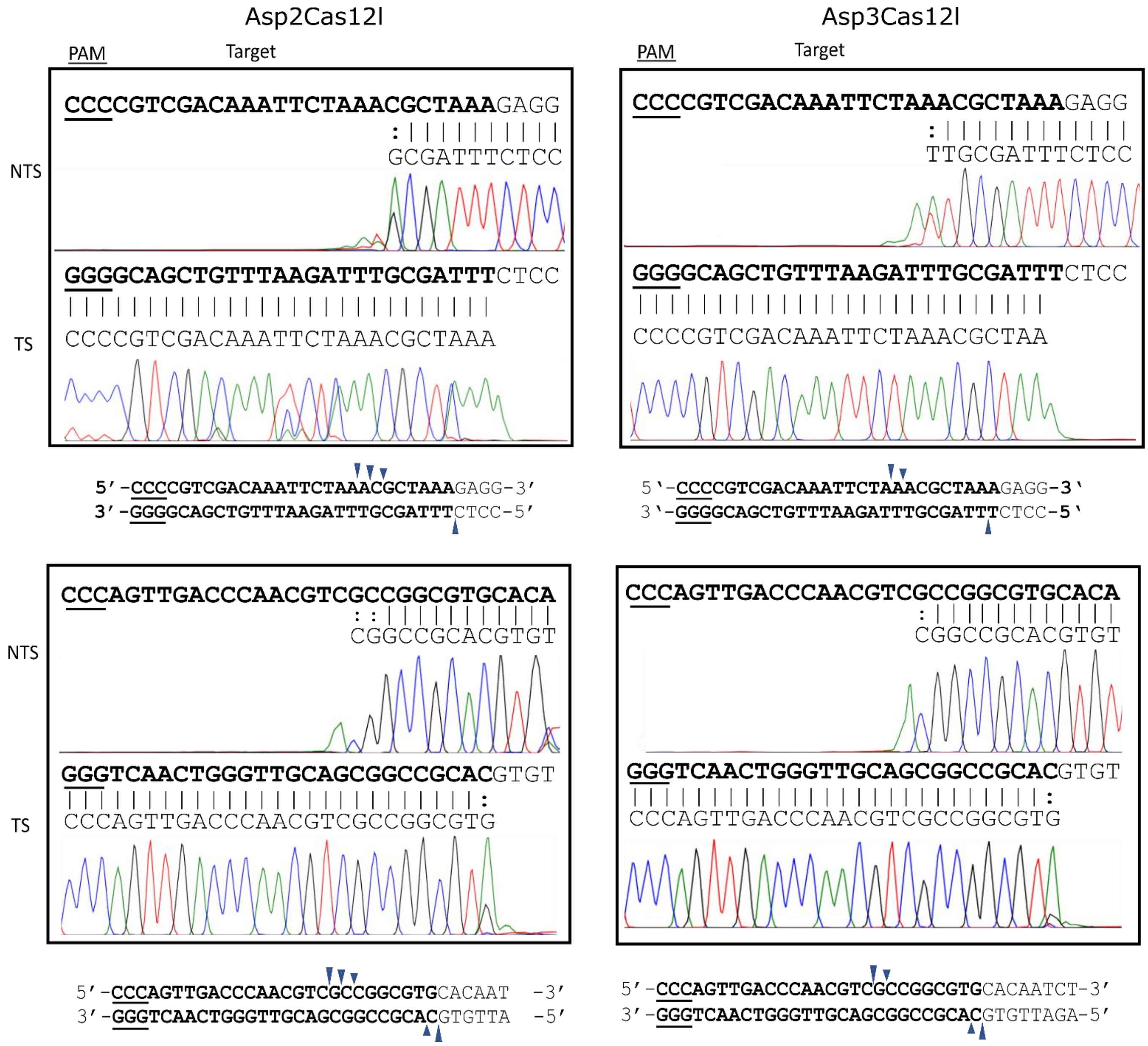

Appendix Figure S4. Run-off Sanger sequencing of Asp2Cas12l & Asp3Cas12l cleaved plasmid substrates. Blue arrows denote the site of cleavage, size of the arrows illustrates prevalence for cleavage at the indicated position.. The target strand is cleaved 23-24 nt 3' of the PAM sequence and the non-target strand is cleaved 15-18 nt 3' of the PAM sequence. A green fluorescent signal is present at the 3' end of the cleavage product in the fluorograms due to the addition of an adenine during sequencing.

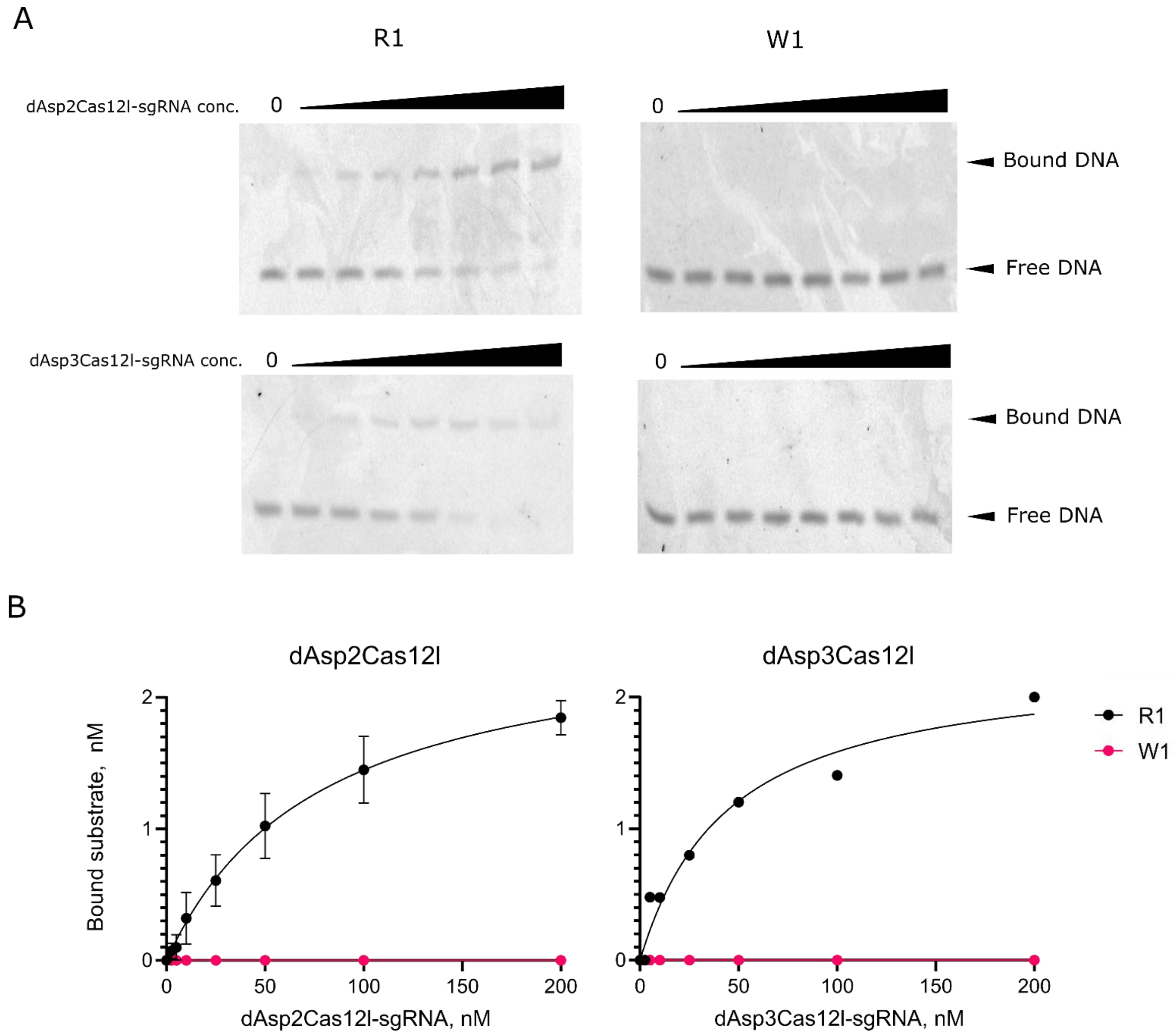

Appendix Figure S5. Electrophoretic mobility shift assays of catalytically inactive or dead (d) Cas12l-sgRNA complexes and fluorescently labelled linear oligoduplex substrates. Under the tested conditions, Cas12l RNP only binds substrates containing the R1 protospacer, which correlates with the observed dsDNA cleavage of R1 and W1 targets as linear substrates. (A) Native PAGE gel images of electrophoretic mobility shift as a function of Cas12l RNP concentration; (B) Densitometric analysis of the fraction of substrate bound to the Cas12l-sgRNA complex fit to a quadratic equation (See Methods & Materials).

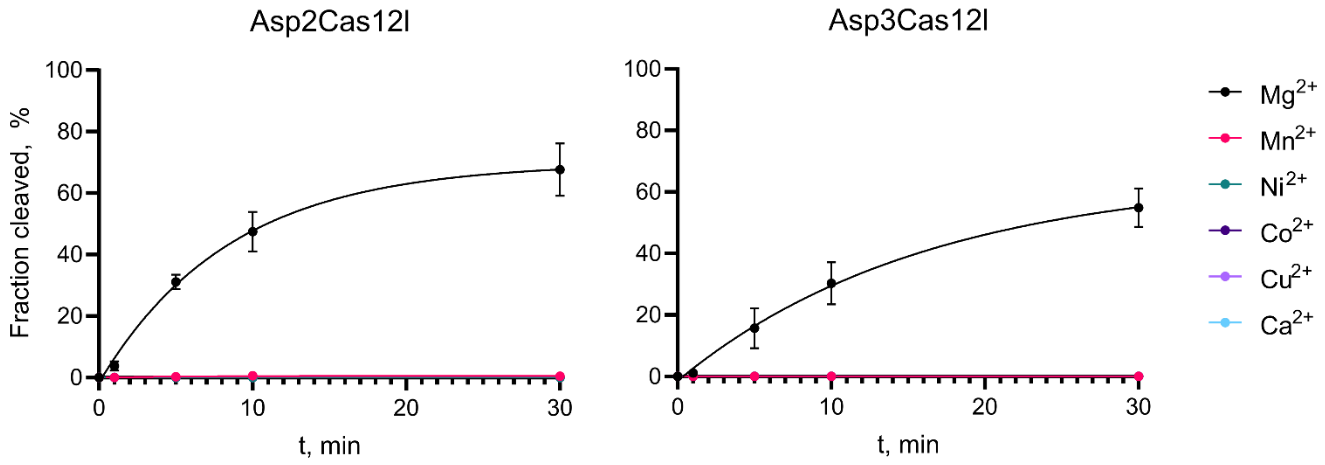

Appendix Figure S6. Metal ion requirements for Cas12l dsDNA cleavage. Only Mg^{2+} metal ions facilitate linear dsDNA cleavage by Asp2Cas12l and Asp3Cas12l. Data presented as mean ± s.d., where n=3 replicates from independent experiments.

A

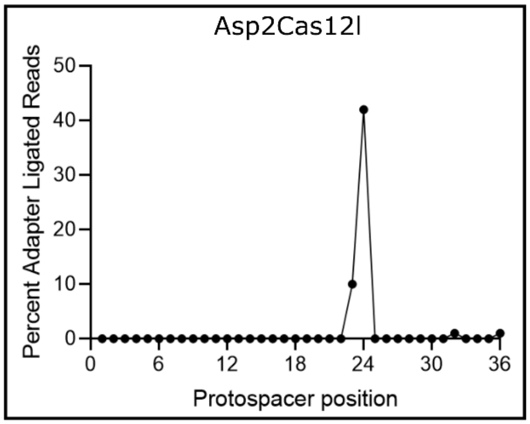

PAM position

Nucleotide	-7	-6	-5	-4	-3	-2	-1
T	0.23	0.25	0.24	0.25	0.00	0.01	0.35
G	0.27	0.28	0.26	0.27	0.00	0.00	0.01
C	0.23	0.24	0.26	0.20	0.99	0.99	0.63
A	0.26	0.24	0.24	0.28	0.01	0.00	0.02
	N	N	N	N	C	C	C

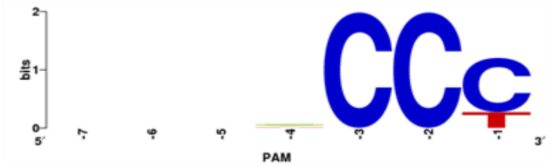

B

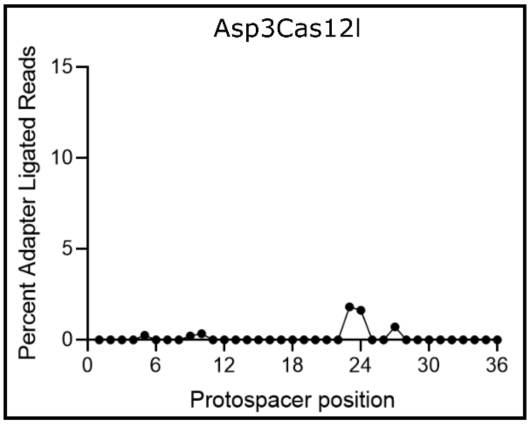

PAM position

Nucleotide	-7	-6	-5	-4	-3	-2	-1
T	0.31	0.30	0.31	0.21	0.00	0.00	0.40
G	0.25	0.16	0.15	0.22	0.00	0.00	0.09
C	0.31	0.29	0.26	0.09	1.00	1.00	0.48
A	0.13	0.25	0.28	0.49	0.00	0.00	0.03
	N	N	N	N	C	C	Y

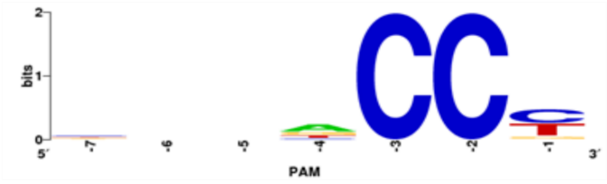

C

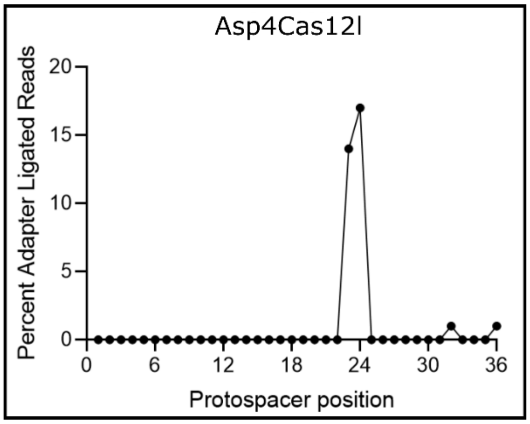

PAM position

Nucleotide	-7	-6	-5	-4	-3	-2	-1
T	0.26	0.27	0.27	0.27	0.01	0.04	0.32
G	0.26	0.25	0.24	0.26	0.00	0.00	0.03
C	0.24	0.25	0.25	0.19	0.97	0.95	0.60
A	0.24	0.23	0.24	0.28	0.02	0.00	0.05
	N	N	N	N	C	C	C

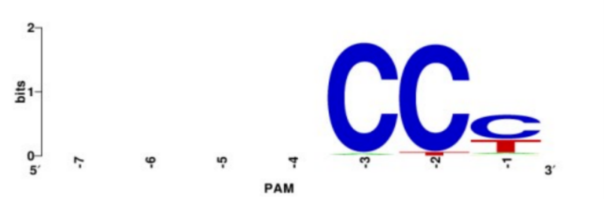

Appendix Figure S7. Cas12l protospacer cleavage position and PAM preferences using purified components. Frequency of adapter ligation as a function of protospacer target position is shown at the left and position frequency weight matrix and weblogo of PAM sequences that supported 7N PAM library dsDNA cleavage is shown at the right. (A) Asp2Cas12l RNP cleavage data; (B) Asp3Cas12l RNP cleavage data; (C) Cas12lδ RNP cleavage data.

A

5'-FAM-accagcaggactacagcttccc**gccttcagaagagggtgcat**tttcagcctttttgtgggtgtacgttttgg-3'

|||||||||||||||||||||||||||||||||||||||||||||||||||||||||||||||||||||||||

3'-tggtcgtcctgatgtcgaagggcggaagtcttctcccacgtaaaagtcggaaaaacacccacatgcaaaacc-ROX-5'

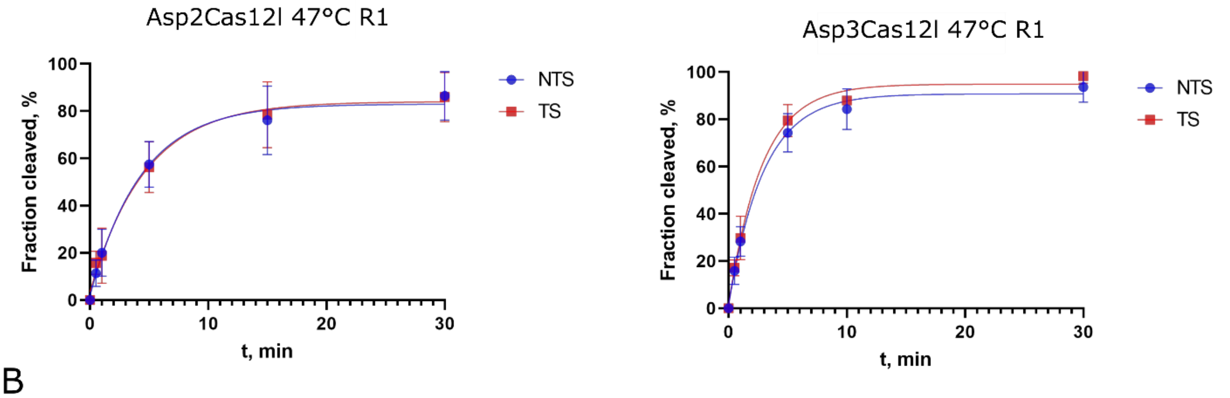

B

5'-FAM-accagcaggactacagcttccc**acagttcgattacctttccc**actcagcctttttgtgggtgtacgttttgg-3'

|||||||||||||||||||||||||||||||||||||||||||||||||||||||||||||||||||||||||

3'-tggtcgtcctgatgtcgaagggtgtcaagctaatggaaagggtgagtcggaaaaacacccacatgcaaaacc-ROX-5'

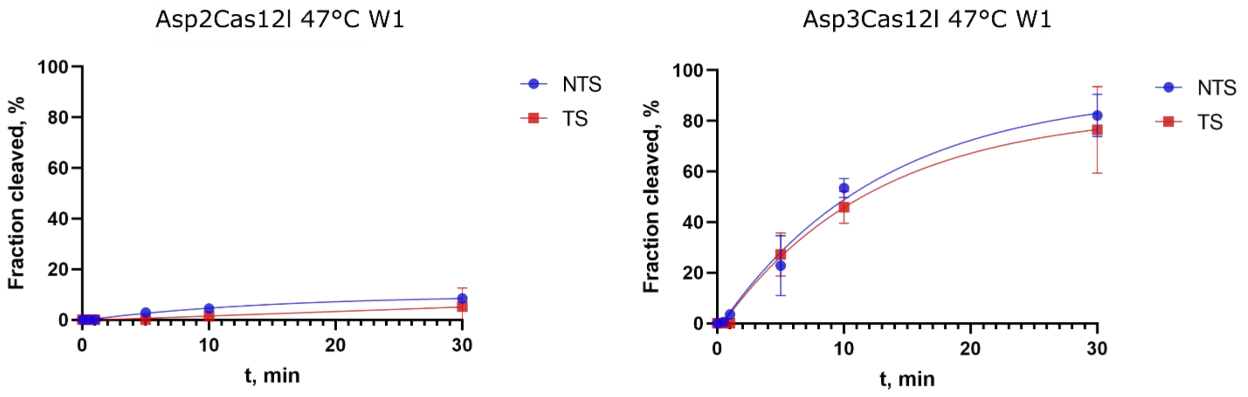

Appendix Figure S8. Asp2Cas12l & Asp3Cas12l target (TS) and non-target DNA strand (NTS) cleavage at elevated temperature. (A) fluorescently labelled (5'-6-FAM depicted in blue & 5'-6-ROX in red) linear oligoduplex dsDNA substrate with 5'-CCC-3' PAM (underlined) and R1 protospacer sequence (bold) used for hydrolysis experiments & cleavage rates of non-target (NTS) and target (TS) DNA strands (B) fluorescently labelled (5'-6-FAM depicted in blue & 5'-6-ROX in red) linear oligoduplex dsDNA substrate with 5'-CCC-3' PAM (underlined) and W1 protospacer sequence (bold) used for hydrolysis experiments & cleavage rates of non-target (NTS) and target (TS) DNA strands. Error bars indicate ± s.d. N=3

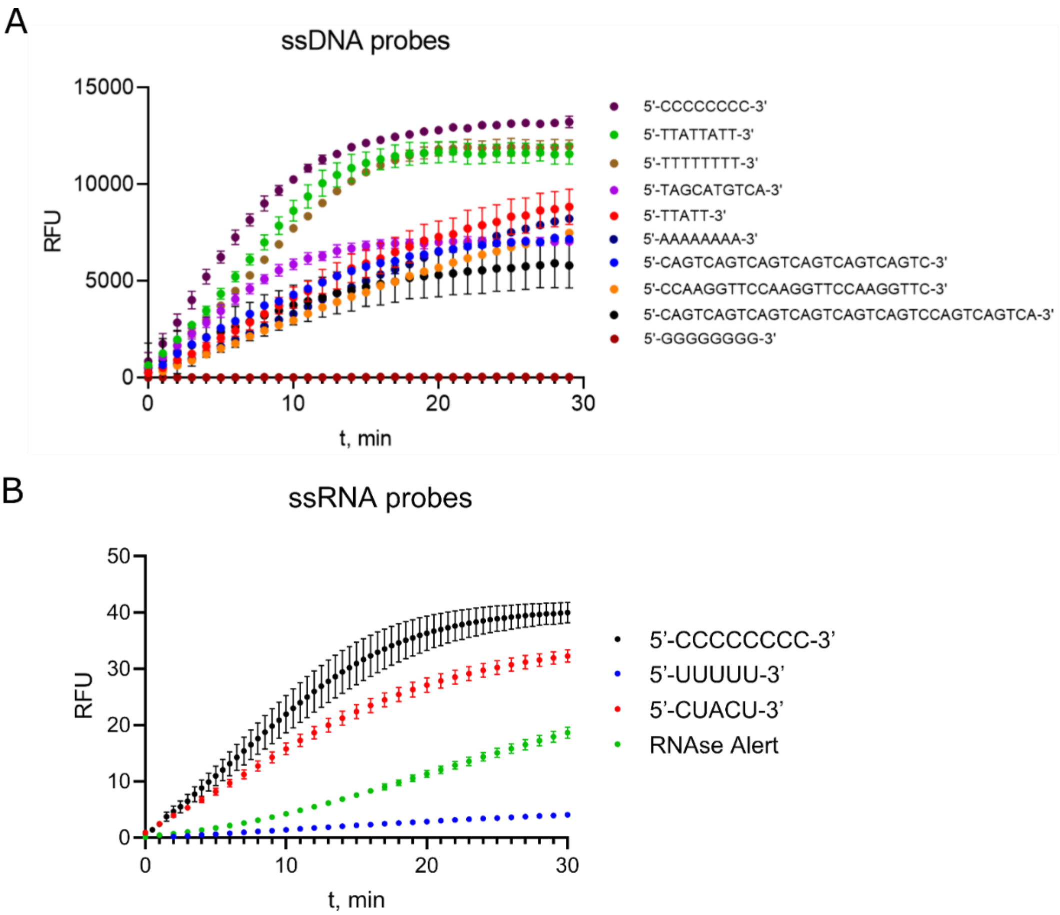

Appendix Figure S9. Effect of probe sequence length and context on Asp2Cas12l trans-degradation. Asp2Cas12l RNP complexes degrade quenched fluorescent ssDNA (A) or ssRNA (B) reporters, with varying efficiencies depending on the probe sequence and length. Fluorescence intensities were normalized against reactions containing only the probe to account for imperfect quenching or degradation of reporters. RFU – relative fluorescence units. Error bars indicate ± s.d., where n = 3 replicates.

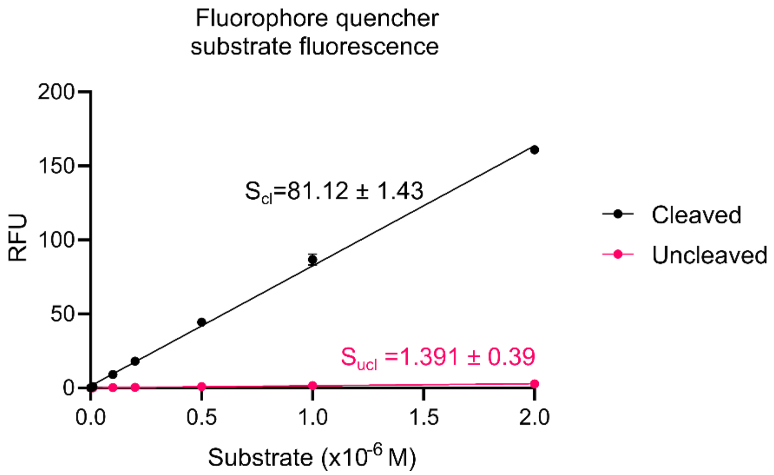

Appendix Figure S10. Standard curves for reporter fluorescence as a function of substrate concentration. Fluorescence versus concentration of uncleaved (red) or fully cleaved (black) substrates. Solid lines were fit using linear regression and the resulting slopes S_{cl} and S_{ucl} are shown with the respective mean ± sd, where n=3. Cl = cleaved, ucl = uncleaved.

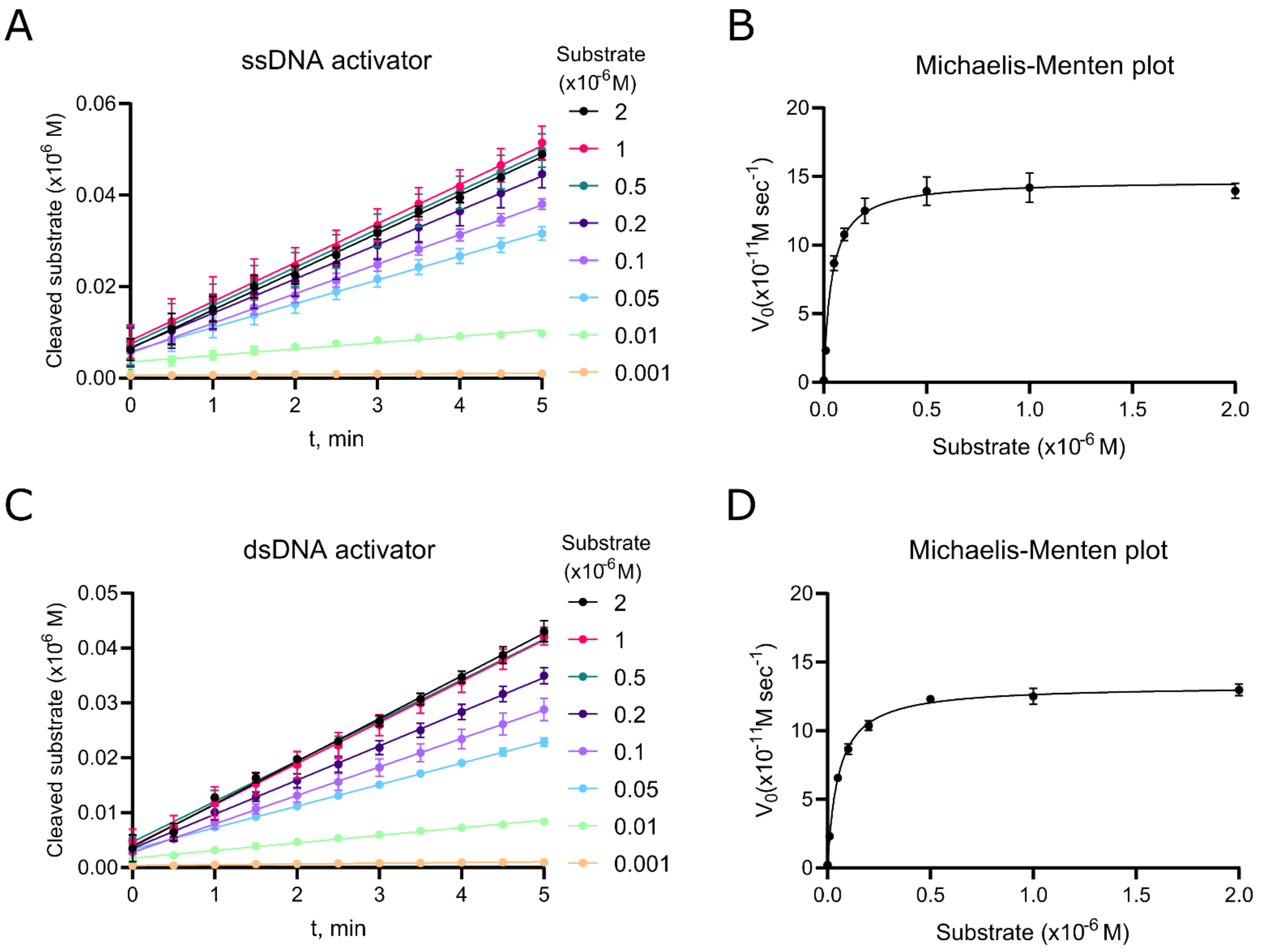

E

Activator	k_{cat} (sec^{-1})	K_M (M)	k_{cat}/K_M (sec^{-1} M^{-1})
ssDNA	1.47 ± 0.05	38.09 ± 6.32 × 10^{-9}	3.87 ± 0.79 × 10^{7}
dsDNA	1.33 ± 0.02	51.37 ± 4.29 × 10^{-9}	2.59 ± 0.47 × 10^{7}

Appendix Figure S11. Michaelis-Menten analysis of LbCas12a collateral ssDNA cleavage activity. Background-subtracted traces and corresponding linear trendlines of cleaved substrate concentration versus time for a (A) ssDNA or (C) dsDNA activator, using 0.1 nM effective LbCas12a-crRNA-activator complex and increasing ssDNA reporter concentration. Error bars indicate ± s.d., where n = 3 replicates. Michaelis-Menten fits for the ssDNA (B) or (D) dsDNA activators. (E) Calculated kinetic constant values, reporting the mean ± s.d., where n = 3 replicates. See Materials & Methods for procedure of analysis.
