# Supplementary material for: A new family of CRISPR‐type V nucleases with C‐rich PAM recognition
Source: EMBO Rep. 2022 Oct 21;23(12):e55481. doi: 10.15252/embr.202255481 (PMC9724661; doi:10.15252/embr.202255481)
Supplement: Supplementary file 2 — Expanded View Figures PDF [file EMBR-23-e55481-s003.pdf]

Expanded View Figures

Figure EV1. Effect of substrate topology and temperature on Cas12l dsDNA cleavage.

A Cas12l dsDNA hydrolysis efficiency varies depending on the target sequence.
B Linear and supercoiled dsDNA substrates with different protospacer sequences (W1 and R1) were interrogated by Asp2Cas12l and Asp3Cas12l RNPs. With a linear topology, only the R1 protospacer target was appreciably cleaved; however, both protospacers were cleaved with similar efficiencies by both proteins when presented in a supercoiled state.
C Effect of reaction temperature on Asp2Cas12l and Asp3Cas12l dsDNA hydrolysis. Optimal temperature for dsDNA cleavage by Asp2Cas12l and Asp3Cas12l RNP complexes is ~50°C.
D Asp3Cas12l dsDNA hydrolysis across various targets at the increased reaction temperature.

Data information: In (A, B, and D), data are presented as mean with individual data points plotted, where $n = 3$ replicates from independent experiments. In (C), individual data points from $n = 3$ replicates from independent experiments are plotted and fitted to a single exponential association curve (solid lines).
Source data are available online for this figure.

 © 2022 The Authors

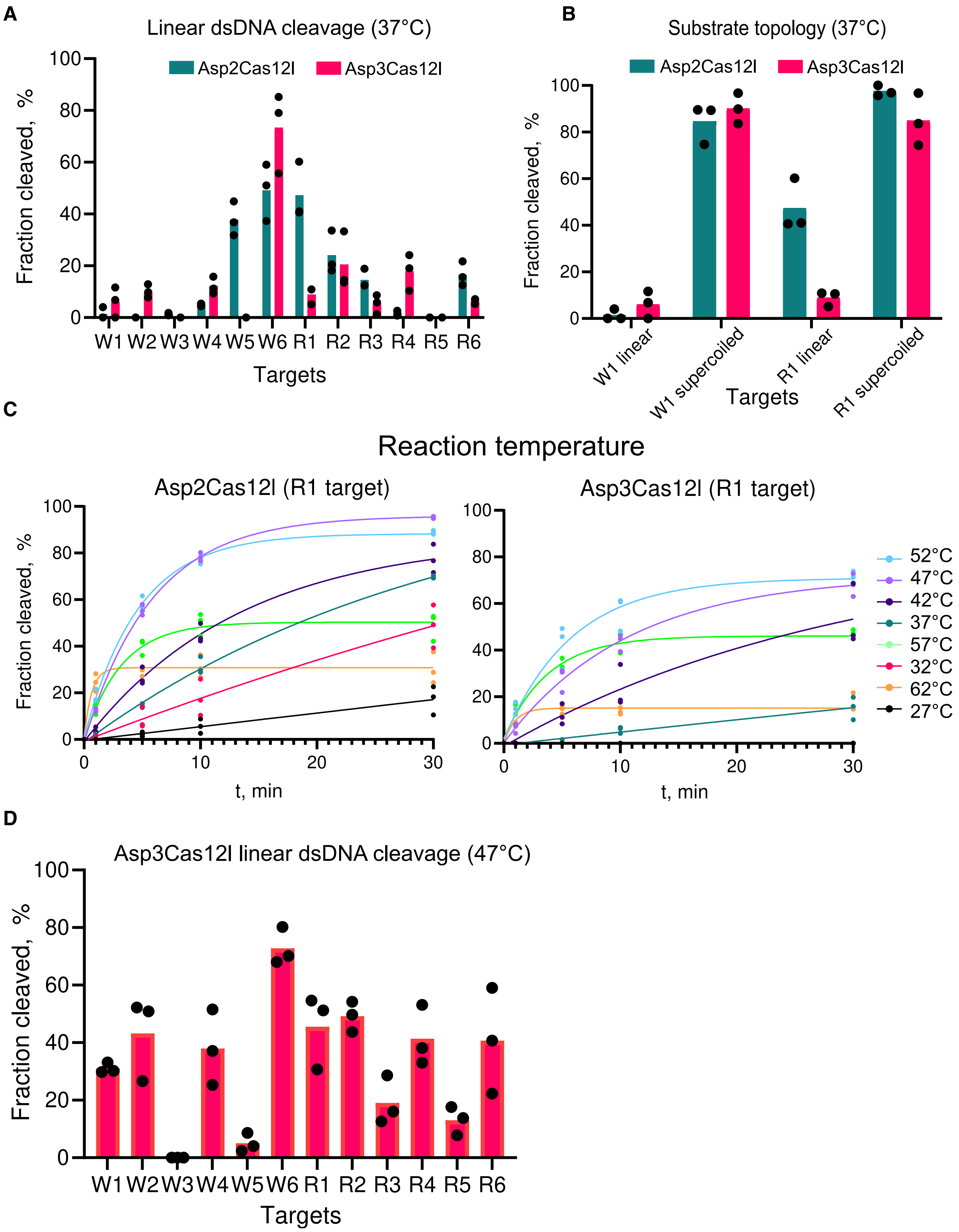
A
Linear dsDNA cleavage (37°C)
Asp2Cas12l
Asp3Cas12l
Fraction cleaved, %
100
80
60
40
20
0
W1 W2 W3 W4 W5 W6 R1 R2 R3 R4 R5 R6
Targets
B
Substrate topology (37°C)
Asp2Cas12l
Asp3Cas12l
Fraction cleaved, %
W1 linear
W1 supercoiled
R1 linear
R1 supercoiled
Targets
C
Reaction temperature
Asp2Cas12l (R1 target)
Asp3Cas12l (R1 target)
Fraction cleaved, %
t, min
52°C
47°C
42°C
37°C
57°C
32°C
62°C
27°C
D
Asp3Cas12l linear dsDNA cleavage (47°C)
Fraction cleaved, %
W1 W2 W3 W4 W5 W6 R1 R2 R3 R4 R5 R6
Targets

Figure EV1.

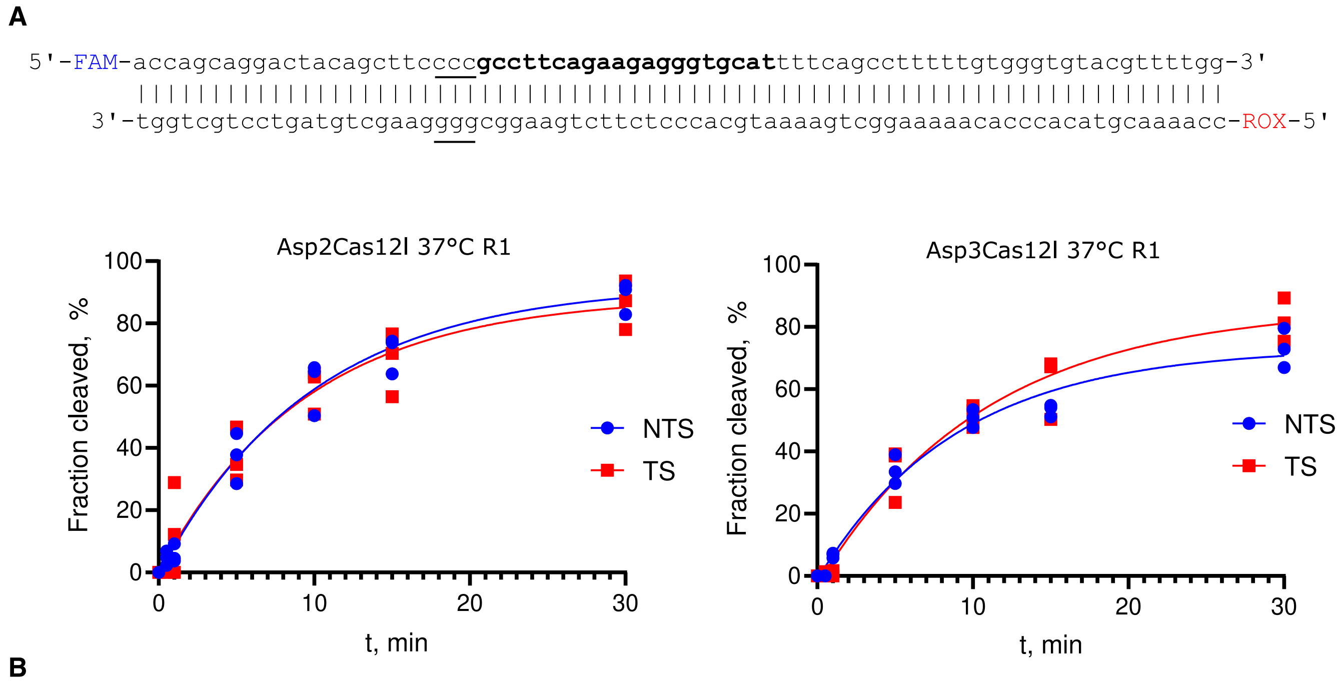

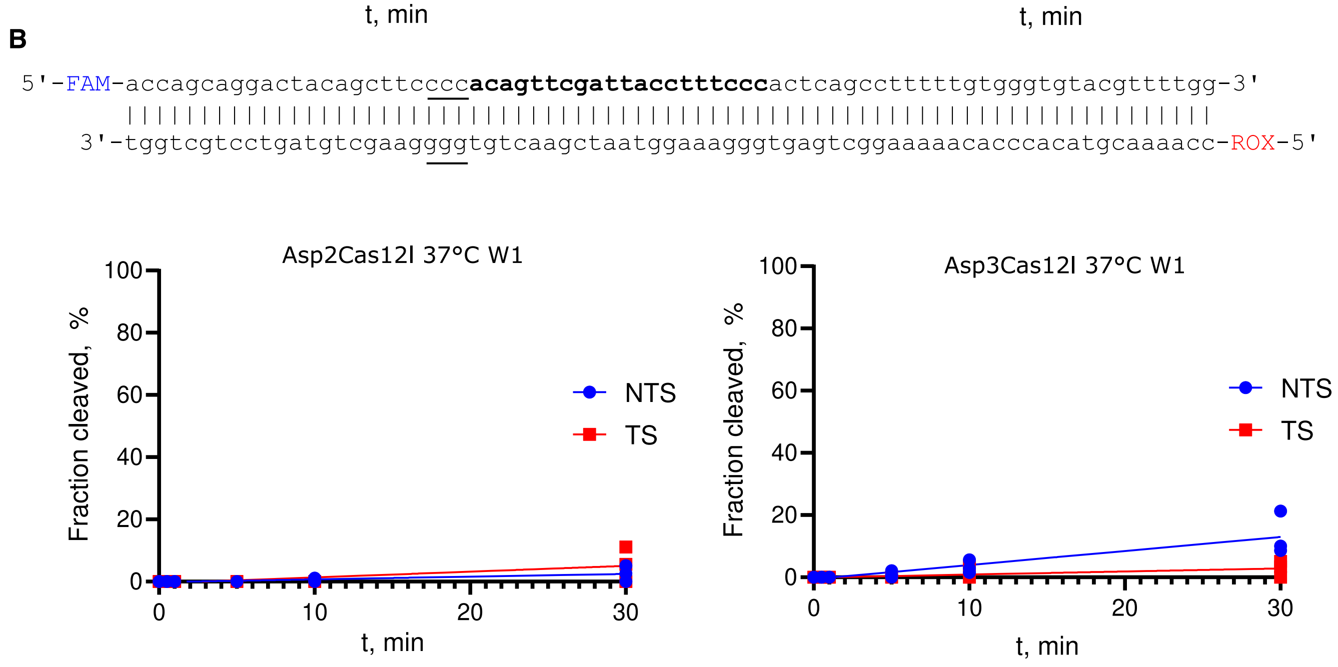

Figure EV2. Asp2Cas12l and Asp3Cas12l cleave both dsDNA strands at similar rates *in vitro*.

A Fluorescently labeled (5′-6-FAM depicted in blue and 5′-6-ROX in red) linear oligoduplex dsDNA substrate with 5′-CCC-3′ PAM (underlined) and R1 protospacer sequence (bold) used for hydrolysis experiments and cleavage rates of nontarget (NTS) and target (TS) DNA strands.

B Fluorescently labeled (5′-6-FAM depicted in blue and 5′-6-ROX in red) linear oligoduplex dsDNA substrate with 5′-CCC-3′ PAM (underlined) and W1 protospacer sequence (bold) used for hydrolysis experiments and cleavage rates of nontarget (NTS) and target (TS) DNA strands.

Data information: Individual data points from $n = 3$ replicates from independent experiments are plotted and fitted to a single exponential association curve (solid lines). Source data are available online for this figure.

 © 2022 The Authors

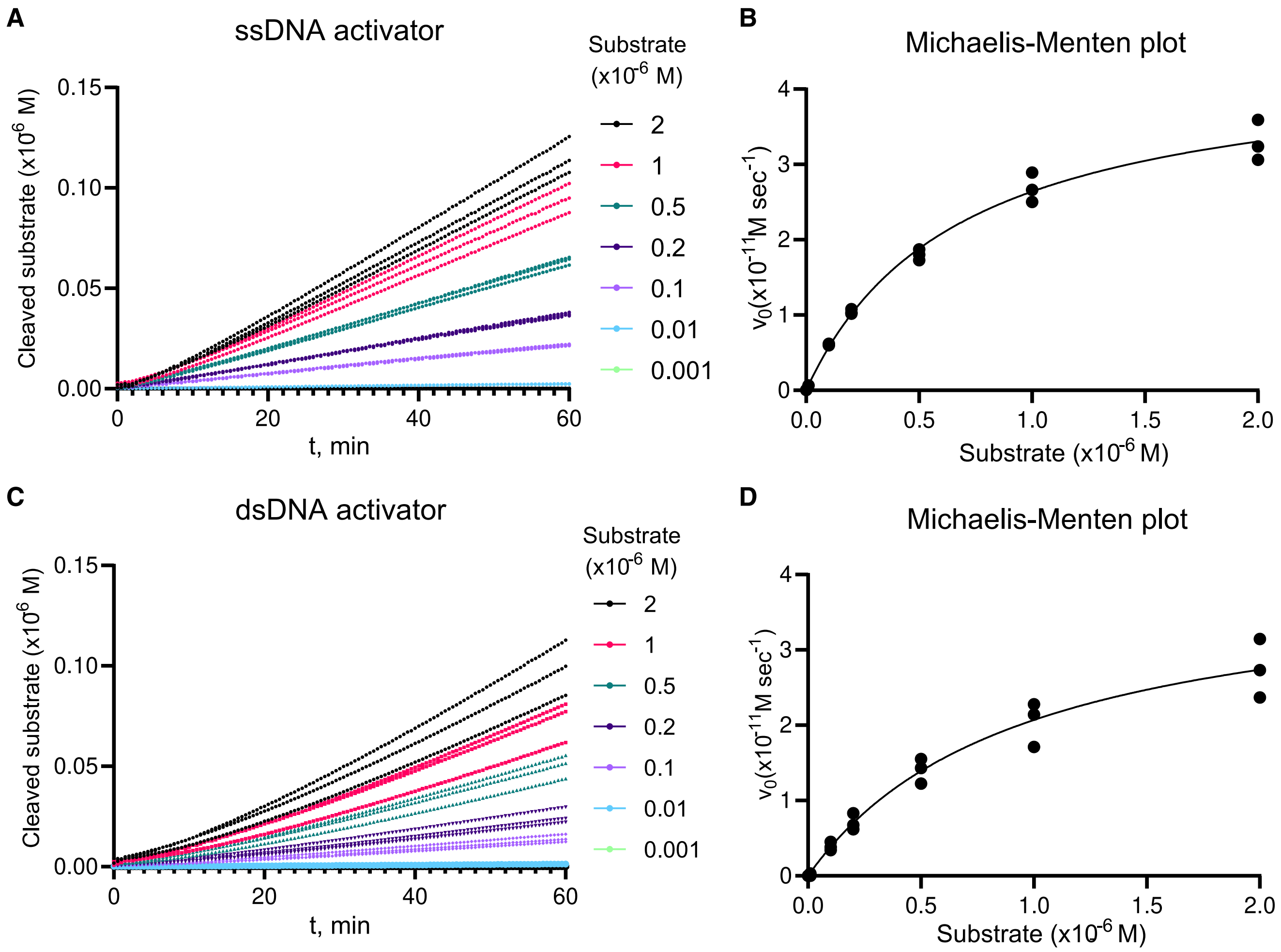

E

Activator	k_{cat} (sec^{-1})	K_M (M)	k_{cat}/K_M (sec^{-1} M^{-1})
ssDNA	0.44 ± 0.03	$0.67 \pm 0.11 \times 10^{-6}$	$6.54 \pm 2.72 \times 10^{5}$
dsDNA	0.40 ± 0.06	$0.95 \pm 0.29 \times 10^{-6}$	$4.21 \pm 2.06 \times 10^{5}$

Figure EV3. Michaelis–Menten analysis of Asp2Cas12l collateral ssDNA cleavage activity.

A Background-subtracted traces and corresponding linear trendlines of cleaved substrate concentration versus time for an ssDNA activator, using 0.1 nM effective Asp2Cas12l-sgRNA-activator complex and increasing ssDNA reporter concentration.

B Michaelis–Menten fits for the ssDNA activator.

C Background-subtracted traces and corresponding linear trendlines of cleaved substrate concentration versus time for a dsDNA activator, using 0.1 nM effective Asp2Cas12l-sgRNA-activator complex and increasing ssDNA reporter concentration.

D Michaelis–Menten fits for the dsDNA activator.

E Calculated kinetic constant values.

Data information: Individual data points from $n = 3$ replicates from independent experiments are plotted. In (E), data are presented as mean ± SD. See Materials and Methods for the procedure of analysis.
Source data are available online for this figure.

© 2022 The Authors
