# Supplementary figures and images for: A new family of CRISPR‐type V nucleases with C‐rich PAM recognition
Source: EMBO Rep. 2022 Oct 21;23(12):e55481. doi: 10.15252/embr.202255481 (PMC9724661; doi:10.15252/embr.202255481)

Asp2Cas12I

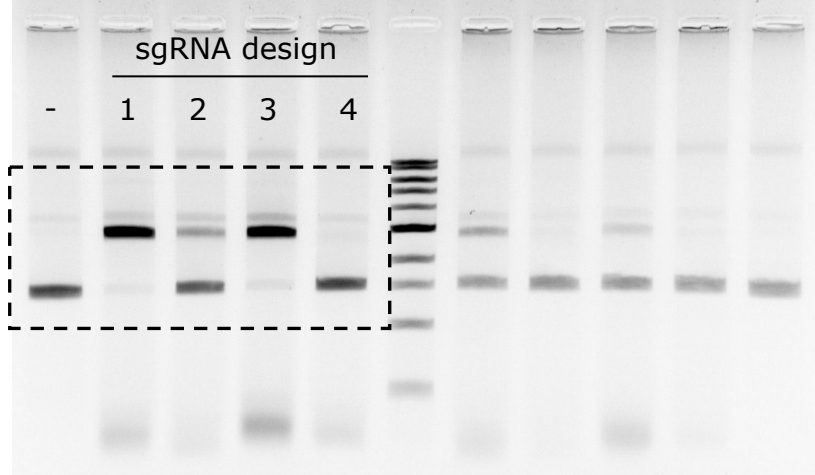

Asp3Cas12I

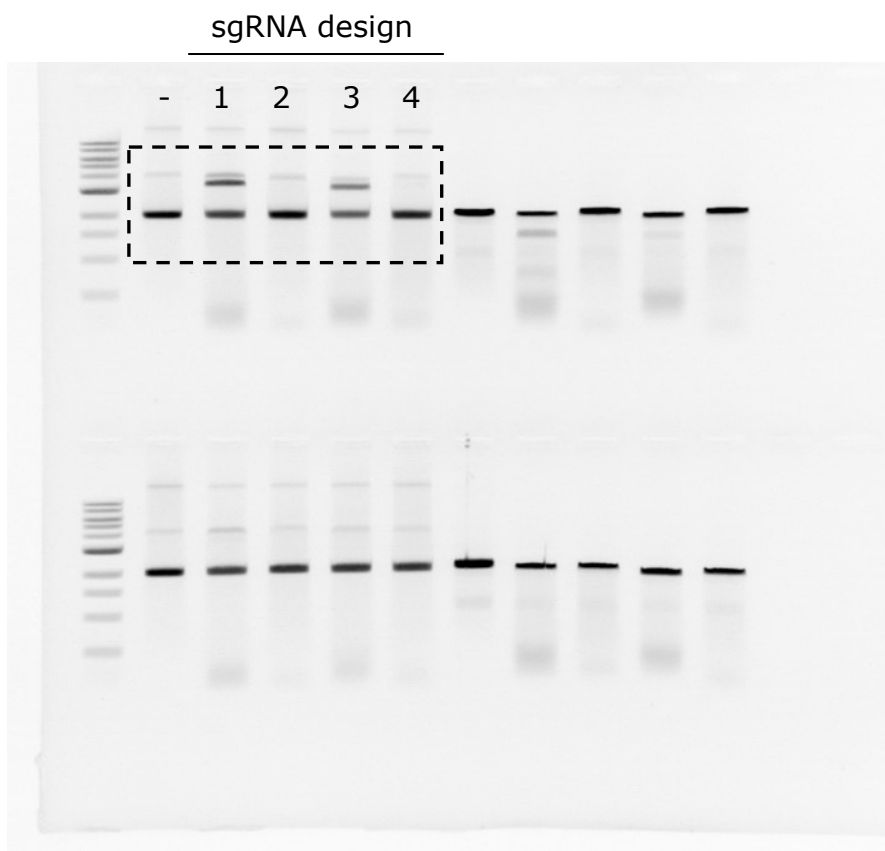

Supplement: Supplementary file 4 — Source Data for Figure 3 [file EMBR-23-e55481-s007.pdf]

Asp2Cas12I

Asp2Cas12I

-Cas12I

wt

D528A

E634A

D803A

wt

D537A

E643A

D796A

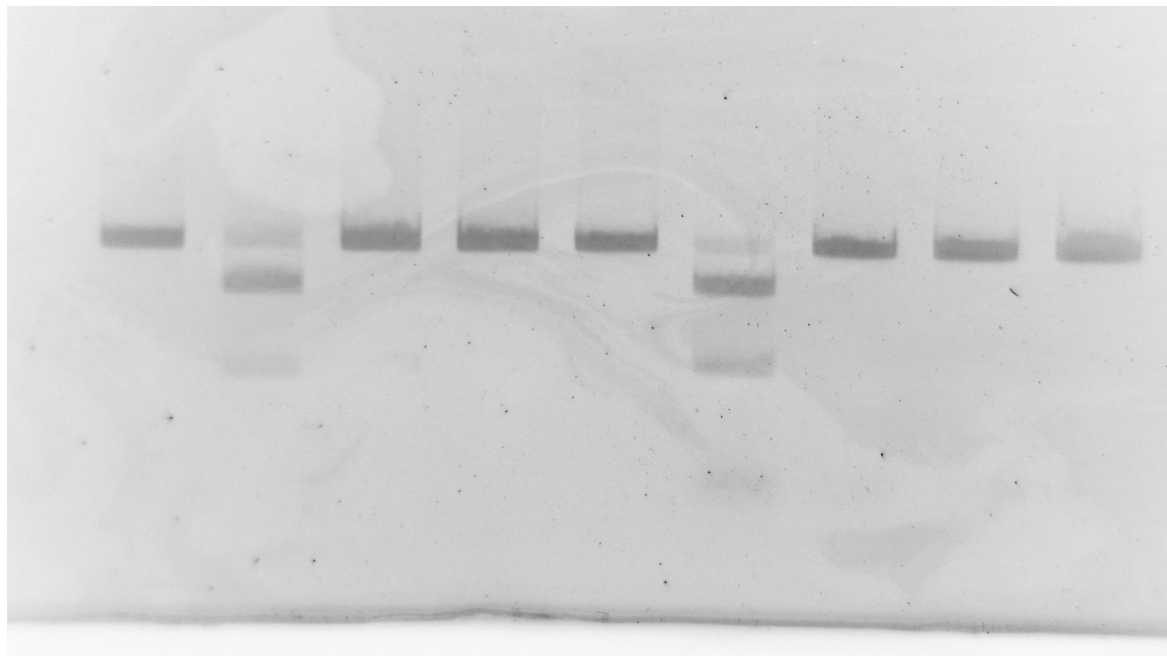

uncut

cut

Supplement: Supplementary file 5 — Source Data for Figure 4 [file EMBR-23-e55481-s005.zip › EMBR_2283_EMBOR202255481T_SDataFig4B.pdf]
