# Supplementary material for: A new family of CRISPR‐type V nucleases with C‐rich PAM recognition
Source: EMBO Rep. 2022 Oct 21;23(12):e55481. doi: 10.15252/embr.202255481 (PMC9724661; doi:10.15252/embr.202255481)

**Asp2Cas12l**	-	+	+	+	+	+	+
gRNA	-	-	+	+	+	+	+
ssDNA act.	-	-	-	+	-	NS	-
dsDNA act.	-	-	-	-	+	-	NS
M13 ssDNA	+	+	+	+	-	+	+

t, min 30 5 30 5 30 5 30 5 30 30 30

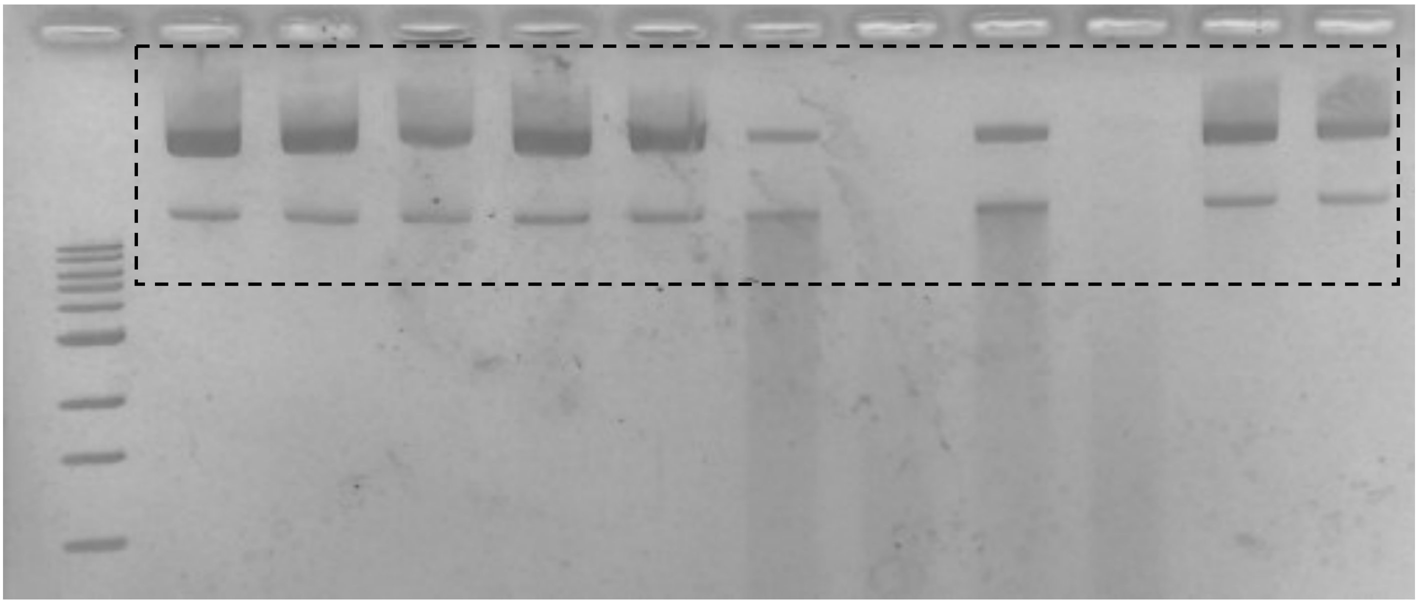

**Asp3Cas12l**	-	+	+	+	+	+	+
gRNA	-	-	+	+	+	+	+
ssDNA act.	-	-	-	+	-	NS	-
dsDNA act.	-	-	-	-	+	-	NS
M13 ssDNA	+	+	+	+	-	+	+

t, min 30 5 30 5 30 5 30 5 30 30 30

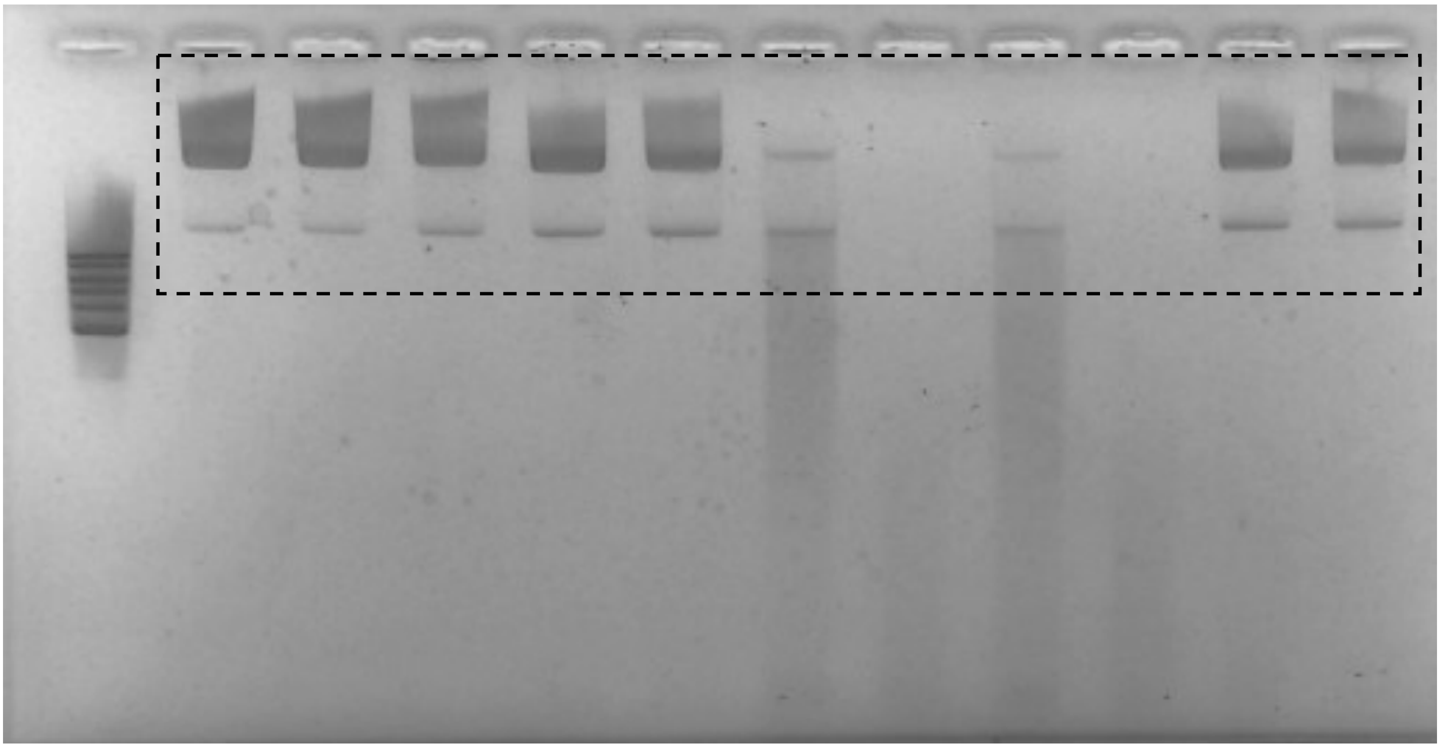

Supplement: Supplementary file 6 — Source Data for Figure 5 [file EMBR-23-e55481-s008.zip › EMBR_2283_EMBOR202255481T_SDataFig5A.pdf]
