# Supplementary material for: A new family of CRISPR‐type V nucleases with C‐rich PAM recognition
Source: EMBO Rep. 2022 Oct 21;23(12):e55481. doi: 10.15252/embr.202255481 (PMC9724661; doi:10.15252/embr.202255481)
Supplement: Supplementary file 9 [file EMBR-23-e55481-s009.pdf]

EMBO reports

A new family of CRISPR-type V nucleases with C-rich PAM recognition

Tomas Urbaitis, Giedrius Gasiunas, Joshua Young, Zhenglin Hou, Sushmitha Paulraj, Egle Godliauskaite, Mantvyda Grusyte, Migle Stitilyte, Monika Jasnauskaite, Megumu Mabuchi, G. Brett Robb, and Virginijus Siksnys
DOI: 10.15252/embr.202255481

Corresponding author(s): Giedrius Gasiunas (giedrius.gasiunas@caszyme.com) , Joshua Young (josh.young@corteva.com)

Review Timeline:

Submission Date:	24th May 22
Editorial Decision:	12th Jul 22
Revision Received:	12th Aug 22
Editorial Decision:	15th Sep 22
Revision Received:	27th Sep 22
Accepted:	29th Sep 22

Editor: Esther Schnapp

Transaction Report:

(Note: With the exception of the correction of typographical or spelling errors that could be a source of ambiguity, letters and reports are not edited. Depending on transfer agreements, referee reports obtained elsewhere may or may not be included in this compilation. Referee reports are anonymous unless the Referee chooses to sign their reports.)

1st Editorial Decision **12th Jul 2022**

Dear Dr. Gasiunas,

Thank you for the submission of your manuscript to EMBO reports. We have now received the full set of referee reports that is pasted below.

As you will see, all referees acknowledge that your findings are interesting. They only have more minor comments, and I think all should be addressed, but please let me know in case you disagree.

I would thus like to invite you to revise your manuscript with the understanding that the referee concerns must be fully addressed and their suggestions taken on board. Please address all referee concerns in a complete point-by-point response. Acceptance of the manuscript will depend on a positive outcome of a second round of review. It is EMBO reports policy to allow a single round of major revision only and acceptance or rejection of the manuscript will therefore depend on the completeness of your responses included in the next, final version of the manuscript.

We realize that it is difficult to revise to a specific deadline. In the interest of protecting the conceptual advance provided by the work, we recommend a revision within 3 months (12th Oct 2022). Please discuss the revision progress ahead of this time with the editor if you require more time to complete the revisions.

IMPORTANT NOTE: we perform an initial quality control of all revised manuscripts before re-review. Your manuscript will FAIL this control and the handling will be DELAYED if the following APPLIES:
1) A data availability section providing access to data deposited in public databases is missing. If you have not deposited any data, please add a sentence to the data availability section that explains that.
2) Your manuscript contains statistics and error bars based on n=2. Please use scatter blots in these cases. No statistics should be calculated if n=2.

When submitting your revised manuscript, please carefully review the instructions that follow below. Failure to include requested items will delay the evaluation of your revision.

1) a .docx formatted version of the manuscript text (including legends for main figures, EV figures and tables). Please make sure that the changes are highlighted to be clearly visible.

2) individual production quality figure files as .eps, .tif, .jpg (one file per figure). See https://wol-prod-cdn.literatumonline.com/pb-assets/embo-site/EMBOPress_Figure_Guidelines_061115-1561436025777.pdf for more info on how to prepare your figures.

3) We replaced Supplementary Information with Expanded View (EV) Figures and Tables that are collapsible/expandable online. A maximum of 5 EV Figures can be typeset. EV Figures should be cited as 'Figure EV1, Figure EV2" etc... in the text and their respective legends should be included in the main text after the legends of regular figures.

- For the figures that you do NOT wish to display as Expanded View figures, they should be bundled together with their legends in a single PDF file called *Appendix*, which should start with a short Table of Content. Appendix figures should be referred to in the main text as: "Appendix Figure S1, Appendix Figure S2" etc. See detailed instructions regarding expanded view here: <https://www.embopress.org/page/journal/14693178/authorguide#expandedview>

- Additional Tables/Datasets should be labeled and referred to as Table EV1, Dataset EV1, etc. Legends have to be provided in a separate tab in case of .xls files. Alternatively, the legend can be supplied as a separate text file (README) and zipped together with the Table/Dataset file.

4) a .docx formatted letter INCLUDING the reviewers' reports and your detailed point-by-point responses to their comments. As part of the EMBO Press transparent editorial process, the point-by-point response is part of the Review Process File (RPF), which will be published alongside your paper.

5) a complete author checklist, which you can download from our author guidelines <https://www.embopress.org/page/journal/14693178/authorguide>. Please insert information in the checklist that is also reflected in the manuscript. The completed author checklist will also be part of the RPF.

6) Please note that all corresponding authors are required to supply an ORCID ID for their name upon submission of a revised manuscript (<https://orcid.org/>). Please find instructions on how to link your ORCID ID to your account in our manuscript tracking system in our Author guidelines
<https://www.embopress.org/page/journal/14693178/authorguide#authorshipguidelines>

7) Before submitting your revision, primary datasets produced in this study need to be deposited in an appropriate public database (see https://www.embopress.org/page/journal/14693178/authorguide#datadeposition). Please remember to provide a

reviewer password if the datasets are not yet public. The accession numbers and database should be listed in a formal "Data Availability" section placed after Materials & Method (see also https://www.embopress.org/page/journal/14693178/authorguide#datadeposition). Please note that the Data Availability Section is restricted to new primary data that are part of this study. * Note - All links should resolve to a page where the data can be accessed. *
If your study has not produced novel datasets, please mention this fact in the Data Availability Section.

8) We would also encourage you to include the source data for figure panels that show essential data. Numerical data should be provided as individual .xls or .csv files (including a tab describing the data). For blots or microscopy, uncropped images should be submitted (using a zip archive if multiple images need to be supplied for one panel). Additional information on source data and instruction on how to label the files are available at <https://www.embopress.org/page/journal/14693178/authorguide#sourcedata>.

9) Our journal also encourages inclusion of *data citations in the reference list* to directly cite datasets that were re-used and obtained from public databases. Data citations in the article text are distinct from normal bibliographical citations and should directly link to the database records from which the data can be accessed. In the main text, data citations are formatted as follows: "Data ref: Smith et al, 2001" or "Data ref: NCBI Sequence Read Archive PRJNA342805, 2017". In the Reference list, data citations must be labeled with "[DATASET]". A data reference must provide the database name, accession number/identifiers and a resolvable link to the landing page from which the data can be accessed at the end of the reference. Further instructions are available at https://www.embopress.org/page/journal/14693178/authorguide#referencesformat

10) Regarding data quantification (see Figure Legends: https://www.embopress.org/page/journal/14693178/authorguide#figureformat)

The following points must be specified in each figure legend:

- the name of the statistical test used to generate error bars and P values,

- the number (n) of independent experiments (please specify technical or biological replicates) underlying each data point,

- the nature of the bars and error bars (s.d., s.e.m.),

- If the data are obtained from n {less than or equal to} 2, use scatter blots showing the individual data points.

Discussion of statistical methodology can be reported in the materials and methods section, but figure legends should contain a basic description of n, P and the test applied.

- Please also include scale bars in all microscopy images.

11) The journal requires a statement specifying whether or not authors have competing interests (defined as all potential or actual interests that could be perceived to influence the presentation or interpretation of an article). In case of competing interests, this must be specified in your disclosure statement. Further information: https://www.embopress.org/competing-interests

We would also welcome the submission of cover suggestions, or motifs to be used by our Graphics Illustrator in designing a cover.

As part of the EMBO publication's Transparent Editorial Process, EMBO reports publishes online a Review Process File (RPF) to accompany accepted manuscripts. This File will be published in conjunction with your paper and will include the referee reports, your point-by-point response and all pertinent correspondence relating to the manuscript.

You are able to opt out of this by letting the editorial office know (emboreports@embo.org). If you do opt out, the Review Process File link will point to the following statement: "No Review Process File is available with this article, as the authors have chosen not to make the review process public in this case."

I look forward to seeing a revised form of your manuscript when it is ready. Please use this link to submit your revision: https://embor.msubmit.net/cgi-bin/main.plex

Yours sincerely,

Esther Schnapp, PhD
Senior Editor
EMBO reports

Referee #1:

General comments;
CRISPR-Cas systems derived from bacteria and archaea have been harnessed for precise gene knockout, knock-in, and correction. Because there are numerous prokaryotes on the Earth, various CRISPR-Cas systems exist and to define new CRISPR-Cas system is still important in the genome editing field. In this manuscript, the authors identified and characterized a new family of type V CRISPR system from Armatimonadetes, and newly classified as Cas12l. The authors revealed that the Cas12l nucleases can induce DNA double strand break with defined sgRNAs at target sites having C-rich PAM sequences and also have collateral non-specific ssDNA and ssRNA cleavage activity. I feel that this study is quite valuable in terms of broadening the knowledge of CRISPR system as well as providing another genome editing tool based on Type V Cas12l targeting C-rich PAM. Overall, this manuscript is straightforward and very informative, which can be suitable for this journal, EMBO Reports. I would suggest a few minor comments that may strengthen this manuscript, as follows.

Specific Comments:
1) It would be better to provide the exact PAM sequences of Cas12l in the abstract. The PAMs seem to be 5'-CCN-3' or 5'-CCY-3'.
In addition, it would be helpful to introduce another Cas nucleases having C-rich PAMs such as type II Nme2Cas9 [PMID: 30581144] just for information.

2) It is necessary to describe clearly about 'Target1' and 'Target2' in Figure 2A. I assume that both targets might be necessary to investigate whether upstream or downstream of spacer are recognized.

3) It is not easy to follow up the sgRNA identification step in Figure 3. I understand that sgRNA1 was derived (linked) from Asp1Cas12l and sgRNA3 from As3Cas12l, but both Asp2Cas12l and Asp3Cas12l showed best DNA cleavage activities with sgRNA1 or sgRNA3. This result means that Asp2Cas12l is less functional with bona-fide sgRNA2, isn't it? The detailed description and discussion are required here for broad readers.
Furthermore, it is necessary to note which sgRNA was used in further experiments.

4) In Figure 5C, both Asp2Cas12l and Asp3Cas12l nucleases showed highest DNA cleavage activities at 52oC, but it seems that the function was lost at 57oC (the saturated cleavage rates were lower at 57oC). I suppose that too high temperature over 57oC might affect AspCas12l conformation, resulting in lower activity. Additional discussion is necessary here. It might be necessary to conduct the same experiments at higher temperature (>57oC).

5) It is necessary to suggest optimal gRNA sequences including the length of spacer (truncated form is better?)

Referee #2:

Urbaitis et al. report the discovery and characterization of a new family of type V CRISPR nucleases, which they propose to name as Cas12l. These nucleases contain the common features of Cas12 nucleases, including a C-terminal RuvC domain, collateral cleavage of ssDNA, and staggered cleavage of target DNA. The major difference is that these nucleases recognize a C-rich PAM compared to the general T-rich PAM for other Cas12 nucleases. These nucleases could be potentially applied for genome editing for C-rich target. In addition, some of these nucleases prefer supercoiled substrates.

Overall, the data presented in this paper look solid to me. The paper is clearly written and easy to follow. Figures are also well prepared. I would be supportive for the publication of this work.

Minor point: Is the substrate in Figure 4B linear dsDNA? Mention it in the figure legend.

Referee #3:

Urbaitis et al. characterized Cas12l, a new member of the Cas12 family of DNA endonucleases. Cas12l is compact and employs a C-rich PAM, unlike previously known Cas12a proteins that tend to have T-rich PAMs. Authors further show that AspCas12l cleaves DNA in vitro and in E coli, and furthermore, has the collateral ssDNA cleavage activity characteristic of many Cas12. Thus, Cas12l adds to the toolbox of Cas12 endonucleases for possible applications.

This work provides several novel aspects on CRISPR-Cas12 that will be valuable to the field. By using a set of bioinformatics tools, authors identified Cas12l as RuvC-containing nucleases with a highly divergent N-terminal domains. Authors then carefully identified the PAM sequence required for Cas12l to function and characterized their in vitro cleavage activity with optimized PAM and sgRNA, and by enzyme kinetics. The experiments are thorough, and the conclusions are sound. It provides a model paper

for others to follow when characterizing a new CRISPR enzymes. I only have several points for clarification.

1. Though Cas12l has the typical DED catalytic motif found in RuvC, it will be interesting to learn its divalent metal requirement, as other Cas12 have shown some varying metal requirement;

2. AspCas12l seems to have lower activity than LbCas12a. Do authors have any speculations as to why this is the case, given they have some ideas about the conserved domains?

3. Line 544, the "first-order rate constant" is technically pseudo-first order with substrate in excess;

4. Can authors clarify in Figure 3B if the four variants were made respectively for Asp2Cas12l and Asp3Cas12l? It appears that the 5' tail is important to cleavage for both enzymes while the sequences of the 5' tail for their respective sgRNA are different.

5. In Figure 4A, it would help readers if the sequence for the target and the non-target strand are colored similarly as the sequence reads above. Should the target strand be 5' instead of 3' of the PAM?

6. In Figure 5, It is not clear which substrates, W# or R#, is linear or supercoiled. Please clarify. Please also specify the temperature used for A and B so it can be compared with Figure 5D;

7. Figure 6A, what happened to the two empty lanes at 30 minutes? Was the M13 DNA completely cleaved? It would be helpful to mark the products;

8. Figure S2, it is not clear what the "ctrl" is. Is it a negative or positive control?

9. Figure S10, please describe how the cleaved substrate were generated. Was it cleaved by an independent DNase or RNase?

POINT-BY-POINT RESPONSE TO REVIEWERS' COMMENTS

Reviewer #1
1. It would be better to provide the exact PAM sequences of Cas12l in the abstract. The PAMs seem to be 5'-CCN-3' or 5'-CCY-3'.
In addition, it would be helpful to introduce another Cas nucleases having C-rich PAMs such as type II Nme2Cas9 [PMID: 30581144] just for information.

REPLY:
Abstract updated to include the exact PAM sequence as suggested. Additional Cas nucleases recognizing a C-rich PAM referenced in the Discussion section.

2. It is necessary to describe clearly about 'Target1' and 'Target2' in Figure 2A. I assume that both targets might be necessary to investigate whether upstream or downstream of spacer are recognized.

REPLY:
Both targets were interrogated in the assay, since T1 and T2 spacers corresponding to Target1 and Target2 were encoded in the Cas12l loci (Figure 2A). Figure legend modified for clarity.

3. It is not easy to follow up the sgRNA identification step in Figure 3. I understand that sgRNA1 was derived (linked) from Asp1Cas12l and sgRNA3 from As3Cas12l, but both Asp2Cas12l and Asp3Cas12l showed best DNA cleavage activities with sgRNA1 or sgRNA3. This result means that Asp2Cas12l is less functional with bona-fide sgRNA2, isn't it? The detailed description and discussion are required here for broad readers.
Furthermore, it is necessary to note which sgRNA was used in further experiments.

REPLY:
Figure 3B shows sgRNA design variants that were constructed for each individual Asp12l ortholog, i.e. the "bona-fide" sgRNAs were derived for each ortholog individually (the full sequences in Figure 3A), only the omission or presence of 5' unstructured region or the 3' terminator-like repeat differed between variants in Figure 3B and 3C. Main text, Figure 3 and legend modified to emphasize.

4. In Figure 5C, both Asp2Cas12l and Asp3Cas12l nucleases showed highest DNA cleavage activities at 52oC, but it seems that the function was lost at 57oC (the saturated cleavage rates were lower at 57oC). I suppose that too high temperature over 57oC might affect AspCas12l conformation, resulting in lower activity. Additional discussion is necessary here. It might be necessary to conduct the same experiments at higher temperature (>57oC).

REPLY:
Indeed, we assume that at temperatures above 52°C the enzymes start to become thermally unstable. We repeated the experiment at 62°C and cleavage activity dropped further corroborating this idea. Figure EV1C (previously Figure 5C) updated with new data.

5. It is necessary to suggest optimal gRNA sequences including the length of spacer (truncated form is better?)

REPLY:
We thank the reviewer for a valid suggestion. Reasoning that previously characterized Cas enzymes prefer 20-24 nt spacers, we tested sgRNAs differing in spacer length between 18-24 nt across several

linear dsDNA substrates and found a 20 nt spacer best facilitates cleavage. See Appendix Figure S3B.

Referee #2:

1. Minor point: Is the substrate in Figure 4B linear dsDNA? Mention it in the figure legend.

REPLY:
The substrate is linear dsDNA. Figure legend updated accordingly.

Referee #3:

1. Though Cas12l has the typical DED catalytic motif found in RuvC, it will be interesting to learn its divalent metal requirement, as other Cas12 have shown some varying metal requirement.

REPLY:
We thank the reviewer for a valid suggestion. We performed linear dsDNA cleavage experiments with various metal ions as shown in Appendix Figure S6. Only Mg^{2+} ions supported dsDNA cleavage.

2. AspCas12l seems to have lower activity than LbCas12a. Do authors have any speculations as to why this is the case, given they have some ideas about the conserved domains?

REPLY:
Even though both AspCas12l and LbCas12a share a conserved split RuvC nuclease domain responsible for nucleic acid cleavage, the current absence of any AspCas12l structural data prevents from making thorough comparisons. However, we can hypothesize that it is caused by a combination of different protein sequences and architecture, as well as a potentially sub-optimal guide RNA design in the case of AspCas12l.

3. Line 544, the "first-order rate constant" is technically pseudo-first order with substrate in excess;

REPLY:
Text modified accordingly.

4. Can authors clarify in Figure 3B if the four variants were made respectively for Asp2Cas12l and Asp3Cas12l? It appears that the 5' tail is important to cleavage for both enzymes while the sequences of the 5' tail for their respective sgRNA are different.

REPLY:
Each of the four individual sgRNA design variants depicted in Figure 3B were constructed for each AspCas12l ortholog. Main text, Figure 3 and legend modified for clarity.
Indeed, the seemingly unstructured and not conserved 5’ region is shown to be essential for dsDNA cleavage in both cases.

5. In Figure 4A, it would help readers if the sequence for the target and the non-target strand are colored similarly as the sequence reads above. Should the target strand be 5' instead of 3' of the PAM?

REPLY:
Figure modified accordingly. Yes, the protospacer sequence on the target strand is technically 5' of the PAM, however the PAM sequence is not shown in the graphic.

6. In Figure 5, It is not clear which substrates, W# or R#, is linear or supercoiled. Please clarify. Please also specify the temperature used for A and B so it can be compared with Figure 5D;

REPLY:
Substrates containing W1 and R1 protospacers in 5B (now Figure EV1B) correspond to the W1 and R1 substrates in 5A (now Figure EV1A). Figure modified to specify reaction temperatures.

7. Figure 6A, what happened to the two empty lanes at 30 minutes? Was the M13 DNA completely cleaved? It would be helpful to mark the products;

REPLY:
The M13 ssDNA was digested completely. To keep Figure 6 (now Figure 5) of appropriate size the gel images are cropped. Raw gel images are provided in the corresponding Source Data file.

8. Figure S2, it is not clear what the "ctrl" is. Is it a negative or positive control?

REPLY:
"ctrl" is a negative control – lysate of *E. coli* not transformed with any plasmid. Figure legend modified to be clearer.

9. Figure S10, please describe how the cleaved substrate were generated. Was it cleaved by an independent DNase or RNase?

REPLY:
Cleaved substrate was generated by incubating with excess of Asp2Cas12l enzyme. Material and Methods section Fluorophore quencher-labelled reporter assays updated to mention this.

1st Revision - Editorial Decision 15th Sep 2022

Dear Dr. Gasiunas

Thank you for the submission of your revised manuscript. We have now received the enclosed report from referee 1 whom I asked to assess your response to all referee comments. Referee 1 is satisfied with the revised manuscript and your responses, and we can therefore in principle proceed with its official acceptance.

However, a few editorial requests still need to be addressed:

- Your manuscript has 5 main figures but is layed out as a full article. Please either add one more main figure or combine the results and discussion sections. The total character count of a short report may not exceed 27.000 characters (excluding materials and methods and references). You can find more information on our article formats in our guide to authors online.

- Please add up to 5 keywords to the ms file.

- Please correct the conflict of interest subheading to "Disclosure and Competing Interest Statement"

- Please remove the author credits from the ms file. We now use CRediT to specify the contributions of each author in the journal submission system instead.

- Please correct the EMBO reports reference style (Harvard style) that can also be found in EndNote. It is no longer numerical but alphabetical.

- The author checklist has not been fully completed. Your main and your EV figures contain error bars, so please answer all questions on statistics in the checklist and send us a completed file.

- Table EV1 needs to be renamed as Dataset EV1. Please add the legend to the first tab of the table.

- In the Appendix table of content, please list the figures individually and add the page number for each figure.

- Please delete the EV Table legend from the ms file.

I attach to this email a related ms file with comments by our data editors. Please address all comments in the final manuscript.

EMBO press papers are accompanied online by A) a short (1-2 sentences) summary of the findings and their significance, B) 2-3 bullet points highlighting key results and C) a synopsis image that is exactly 550 pixels wide and 200-600 pixels high (the height is variable). You can either show a model or key data in the synopsis image. Please note that text needs to be readable at the final size. Please send us this information along with the revised manuscript.

I look forward to seeing a final version of your manuscript as soon as possible. Please use this link to submit your revision: https://embor.msubmit.net/cgi-bin/main.plex

Best regards,
Esther

Esther Schnapp, PhD
Senior Editor
EMBO reports

Referee #1:

The authors have mostly answered the issues I raised in the earlier review. I recommend the publication of this revised version.

The authors have addressed all minor editorial requests.

Dr. Giedrius Gasiunas
CasZyme
Sauletekio 7c
Vilnius, Vilnius LT-10257
Lithuania

Dear Dr. Gasiunas,

I am very pleased to accept your manuscript for publication in the next available issue of EMBO reports. Thank you for your contribution to our journal.

At the end of this email I include important information about how to proceed. Please ensure that you take the time to read the information and complete and return the necessary forms to allow us to publish your manuscript as quickly as possible.

As part of the EMBO publication's Transparent Editorial Process, EMBO reports publishes online a Review Process File to accompany accepted manuscripts. As you are aware, this File will be published in conjunction with your paper and will include the referee reports, your point-by-point response and all pertinent correspondence relating to the manuscript.

If you do NOT want this File to be published, please inform the editorial office within 2 days, if you have not done so already, otherwise the File will be published by default [contact: emboreports@embo.org]. If you do opt out, the Review Process File link will point to the following statement: "No Review Process File is available with this article, as the authors have chosen not to make the review process public in this case." Please note that the author checklist will still be published even if you opt out of the transparent process.

Thank you again for your contribution to EMBO reports and congratulations on a successful publication. Please consider us again in the future for your most exciting work.

Yours sincerely,

Esther Schnapp, PhD
Senior Editor
EMBO reports

****

THINGS TO DO NOW:

Please note that you will be contacted by Wiley Author Services to complete licensing and payment information. The required 'Page Charges Authorization Form' is available here: https://www.embopress.org/pb-assets/embo-site/er_apc.pdf - please download and complete the form and return to embopressproduction@wiley.com

EMBO Press participates in many Publish and Read agreements that allow authors to publish Open Access with reduced/no publication charges. Check your eligibility: https://authorservices.wiley.com/author-resources/Journal-Authors/open-access/affiliation-policies-payments/index.html

You will receive proofs by e-mail approximately 2-3 weeks after all relevant files have been sent to our Production Office; you should return your corrections within 2 days of receiving the proofs.

Please inform us if there is likely to be any difficulty in reaching you at the above address at that time. Failure to meet our deadlines may result in a delay of publication, or publication without your corrections.

All further communications concerning your paper should quote reference number EMBOR-2022-55481V3 and be addressed to emboreports@wiley.com.

Should you be planning a Press Release on your article, please get in contact with emboreports@wiley.com as early as possible, in order to coordinate publication and release dates.

EMBO Press Author Checklist

Corresponding Author Name: Giedrius Gasiunas	
Journal Submitted to: EMBO Reports	
Manuscript Number: EMBOR-2022-55481	

USEFUL LINKS FOR COMPLETING THIS FORM
The EMBO Journal - Author Guidelines
EMBO Reports - Author Guidelines
Molecular Systems Biology - Author Guidelines
EMBO Molecular Medicine - Author Guidelines

Reporting Checklist for Life Science Articles (updated January 2022)
This checklist is adapted from Materials Design Analysis Reporting (MDAR) Checklist for Authors. MDAR establishes a minimum set of requirements in transparent reporting in the life sciences (see Statement of Task: 10.31222/osf.io/9sm4x). Please follow the journal's guidelines in preparing your manuscript.
Please note that a copy of this checklist will be published alongside your article.

Abridged guidelines for figures
1. Data
The data shown in figures should satisfy the following conditions:
- the data were obtained and processed according to the field's best practice and are presented to reflect the results of the experiments in an accurate and unbiased manner.
- ideally, figure panels should include only measurements that are directly comparable to each other and obtained with the same assay.
- plots include clearly labeled error bars for independent experiments and sample sizes. Unless justified, error bars should not be shown for technical replicates.
- if n<5, the individual data points from each experiment should be plotted. Any statistical test employed should be justified.
- Source Data should be included to report the data underlying figures according to the guidelines set out in the authorship guidelines on Data Presentation.

2. Captions
Each figure caption should contain the following information, for each panel where they are relevant:
- a specification of the experimental system investigated (eg cell line, species name).
- the assay(s) and method(s) used to carry out the reported observations and measurements.
- an explicit mention of the biological and chemical entity(ies) that are being measured.
- an explicit mention of the biological and chemical entity(ies) that are altered/varied/perturbed in a controlled manner.
- the exact sample size (n) for each experimental group/condition, given as a number, not a range;
- a description of the sample collection allowing the reader to understand whether the samples represent technical or biological replicates (including how many animals, litters, cultures, etc.).
- a statement of how many times the experiment shown was independently replicated in the laboratory.
- definitions of statistical methods and measures:
  - common tests, such as t-test (please specify whether paired vs. unpaired), simple χ2 tests, Wilcoxon and Mann-Whitney tests, can be unambiguously identified by name only, but more complex techniques should be described in the methods section;
  - are tests one-sided or two-sided?
  - are there adjustments for multiple comparisons?
  - exact statistical test results, e.g., P values = x but not P values < x;
  - definition of 'center values' as median or average;
  - definition of error bars as s.d. or s.e.m.

Please complete ALL of the questions below.
Select "Not Applicable" only when the requested information is not relevant for your study.

Materials

Newly Created Materials	Information included in the manuscript?	In which section is the information available? (Reagents and Tools Table, Materials and Methods, Figures, Data Availability Section)
New materials and reagents need to be available; do any restrictions apply?	Not Applicable	

Antibodies	Information included in the manuscript?	In which section is the information available? (Reagents and Tools Table, Materials and Methods, Figures, Data Availability Section)
For **antibodies** provide the following information:<br>- Commercial antibodies: RRID (if possible) or supplier name, catalogue number and or/clone number<br>- Non-commercial: RRID or citation	Not Applicable	

DNA and RNA sequences	Information included in the manuscript?	In which section is the information available? (Reagents and Tools Table, Materials and Methods, Figures, Data Availability Section)
**Short novel DNA or RNA including primers, probes**: provide the sequences.	Yes	Table EV1

Cell materials	Information included in the manuscript?	In which section is the information available? (Reagents and Tools Table, Materials and Methods, Figures, Data Availability Section)
**Cell lines:** Provide species information, strain. Provide accession number in repository **OR** supplier name, catalog number, clone number, and/**OR** RRID.	Not Applicable	
**Primary cultures:** Provide species, strain, sex of origin, genetic modification status.	Not Applicable	
Report if the cell lines were recently **authenticated** (e.g., by STR profiling) and tested for mycoplasma contamination.	Not Applicable	

Experimental animals	Information included in the manuscript?	In which section is the information available? (Reagents and Tools Table, Materials and Methods, Figures, Data Availability Section)
**Laboratory animals or Model organisms:** Provide species, strain, sex, age, genetic modification status. Provide accession number in repository **OR** supplier name, catalog number, clone number, **OR** RRID.	Not Applicable	
**Animal observed in or captured from the field:** Provide species, sex, and age where possible.	Not Applicable	
Please detail **housing and husbandry conditions**.	Not Applicable	

Plants and microbes	Information included in the manuscript?	In which section is the information available? (Reagents and Tools Table, Materials and Methods, Figures, Data Availability Section)
**Plants:** provide species and strain, ecotype and cultivar where relevant, unique accession number if available, and source (including location for collected wild specimens).	Not Applicable	
**Microbes:** provide species and strain, unique accession number if available, and source.	Not Applicable	

Human research participants	Information included in the manuscript?	In which section is the information available? (Reagents and Tools Table, Materials and Methods, Figures, Data Availability Section)
If collected and within the bounds of privacy constraints report on age, sex and gender or ethnicity for all study participants.	Not Applicable	

Core facilities	Information included in the manuscript?	In which section is the information available? (Reagents and Tools Table, Materials and Methods, Figures, Data Availability Section)
If your work benefited from core facilities, was their service mentioned in the acknowledgments section?	Not Applicable	

Design

Study protocol	Information included in the manuscript?	In which section is the information available? (Reagents and Tools Table, Materials and Methods, Figures, Data Availability Section)
If study protocol has been **pre-registered, provide DOI in the manuscript**. For clinical trials, provide the trial registration number **OR** cite DOI.	Not Applicable	
Report the **clinical trial registration number** (at ClinicalTrials.gov or equivalent), where applicable.	Not Applicable	

Laboratory protocol	Information included in the manuscript?	In which section is the information available? (Reagents and Tools Table, Materials and Methods, Figures, Data Availability Section)
Provide DOI OR other citation details if **external detailed step-by-step protocols** are available.	Not Applicable	

Experimental study design and statistics	Information included in the manuscript?	In which section is the information available? (Reagents and Tools Table, Materials and Methods, Figures, Data Availability Section)
Include a statement about **sample size** estimate even if no statistical methods were used.	Yes	Figure legends
Were any steps taken to minimize the effects of subjective bias when allocating animals/samples to treatment (e.g. **randomization procedure**)? If yes, have they been described?	Not Applicable	No randomization steps were taken
Include a statement about **blinding** even if no blinding was done.	Not Applicable	No blinding was done
Describe **inclusion/exclusion criteria** if samples or animals were excluded from the analysis. Were the criteria pre-established?<br><br>If sample or data points were omitted from analysis, report if this was due to attrition or intentional exclusion and provide justification.	Not Applicable	No samples were omitted
For every figure, are **statistical tests** justified as appropriate? Do the data meet the assumptions of the tests (e.g., normal distribution)? Describe any methods used to assess it. Is there an estimate of variation within each group of data? Is the variance similar between the groups that are being statistically compared?	Not Applicable	No statistical tests were performed in this study

Sample definition and in-laboratory replication	Information included in the manuscript?	In which section is the information available? (Reagents and Tools Table, Materials and Methods, Figures, Data Availability Section)
In the figure legends: state number of times the experiment was **replicated** in laboratory.	Yes	Figure legends
In the figure legends: define whether data describe **technical or biological replicates**.	Yes	Figure legends

Ethics

Ethics	Information included in the manuscript?	In which section is the information available? (Reagents and Tools Table, Materials and Methods, Figures, Data Availability Section)
Studies involving **human participants**: State details of **authority granting ethics approval** (IRB or equivalent committee(s), provide reference number for approval.	Not Applicable	
Studies involving **human participants**: Include a statement confirming that **informed consent** was obtained from all subjects and that the experiments conformed to the principles set out in the WMA Declaration of Helsinki and the Department of Health and Human Services Belmont Report.	Not Applicable	
Studies involving **human participants:** For publication of **patient photos**, include a statement confirming that consent to publish was obtained.	Not Applicable	
Studies involving experimental **animals**: State details of **authority granting ethics approva**l (IRB or equivalent committee(s), provide reference number for approval. Include a statement of compliance with ethical regulations.	Not Applicable	
Studies involving **specimen and field samples:** State if relevant **permits** obtained, provide details of authority approving study; if none were required, explain why.	Not Applicable	

Dual Use Research of Concern (DURC)	Information included in the manuscript?	In which section is the information available? (Reagents and Tools Table, Materials and Methods, Figures, Data Availability Section)
Could your study fall under dual use research restrictions? Please check biosecurity documents and list of **select agents and toxins** (CDC): https://www.selectagents.gov/sat/list.htm	Not Applicable	
If you used a select agent, is the security level of the lab appropriate and reported in the manuscript?	Not Applicable	
If a study is subject to dual use research of concern regulations, is the name of the **authority granting approval and reference number** for the regulatory approval provided in the manuscript?	Not Applicable	

Reporting

The MDAR framework recommends adoption of discipline-specific guidelines, established and endorsed through community initiatives. Journals have their own policy about requiring specific guidelines and recommendations to complement MDAR.

Adherence to community standards	Information included in the manuscript?	In which section is the information available? (Reagents and Tools Table, Materials and Methods, Figures, Data Availability Section)
State if relevant guidelines or checklists (e.g., **ICMJE, MIBBI, ARRIVE, PRISMA**) have been followed or provided.	Not Applicable	
For **tumor marker prognostic studies**, we recommend that you follow the **REMARK** reporting guidelines (see link list at top right). See author guidelines, under 'Reporting Guidelines'. Please confirm you have followed these guidelines.	Not Applicable	
For **phase II and III randomized controlled trials**, please refer to the **CONSORT** flow diagram (see link list at top right) and submit the CONSORT checklist (see link list at top right) with your submission. See author guidelines, under 'Reporting Guidelines'. Please confirm you have submitted this list.	Not Applicable	

Data Availability

Data availability	Information included in the manuscript?	In which section is the information available? (Reagents and Tools Table, Materials and Methods, Figures, Data Availability Section)
Have **primary datasets** been deposited according to the journal's guidelines (see 'Data Deposition' section) and the respective accession numbers provided in the Data Availability Section?	Yes	Data Availability Section
Were **human clinical and genomic datasets** deposited in a public access-controlled repository in accordance to ethical obligations to the patients and to the applicable consent agreement?	Not Applicable	
Are **computational models** that are central and integral to a study available without restrictions in a machine-readable form? Were the relevant accession numbers or links provided?	Not Applicable	
If publicly available data were reused, provide the respective **data citations in the reference list**.	Not Applicable	
